# Synthesis of α,β-unsaturated esters via a chemo-enzymatic chain elongation approach by combining carboxylic acid reduction and Wittig reaction

**DOI:** 10.3762/bjoc.11.243

**Published:** 2015-11-19

**Authors:** Yitao Duan, Peiyuan Yao, Yuncheng Du, Jinhui Feng, Qiaqing Wu, Dunming Zhu

**Affiliations:** 1 National Engineering Laboratory for Industrial Enzymes and Tianjin Engineering Center for Biocatalytic Technology, Tianjin Institute of Industrial Biotechnology, Chinese Academy of Sciences, Tianjin 300308, P. R. China

**Keywords:** α,β-unsaturated esters, carboxylic acid reductase, chemoenzymatic synthesis, reduction, Wittig reaction

## Abstract

α,β-Unsaturated esters are versatile building blocks for organic synthesis and of significant importance for industrial applications. A great variety of synthetic methods have been developed, and quite a number of them use aldehydes as precursors. Herein we report a chemo-enzymatic chain elongation approach to access α,β-unsaturated esters by combining an enzymatic carboxylic acid reduction and Wittig reaction. Recently, we have found that *Mycobacterium* sp. was able to reduce phenylacetic acid (**1a**) to 2-phenyl-1-ethanol (**1c**) and two sequences in the *Mycobacterium* sp. genome had high identity with the carboxylic acid reductase (CAR) gene from *Nocardia iowensis*. These two putative CAR genes were cloned, overexpressed in *E. coli* and one of two proteins could reduce **1a**. The recombinant CAR was purified and characterized. The enzyme exhibited high activity toward a variety of aromatic and aliphatic carboxylic acids, including ibuprofen. The *Mycobacterium* CAR catalyzed carboxylic acid reduction to give aldehydes, followed by a Wittig reaction to afford the products α,β-unsaturated esters with extension of two carbon atoms, demonstrating a new chemo-enzymatic method for the synthesis of these important compounds.

## Introduction

α,β-Unsaturated esters are versatile building blocks for organic synthesis and of significant importance for industrial applications [1–13]. A great variety of synthetic methods have been developed to access α,β-unsaturated esters [14–24]. One popular approach is the Wittig reaction which produces α,β-unsaturated esters with two more carbon atoms [25]. While fatty acids are abundant from natural resources, aromatic carboxylic acids could be prepared by the degradation of lignin, an unused and abundant component of biomass, although the effective methods for the degradation of lignin need to be developed. These carboxylic acids could be reduced to their corresponding aldehydes, which can be used as starting materials for Wittig reaction. The combination of carboxylic acid reduction and Wittig reaction would offer a new approach for the production of bio-based α,β-unsaturated esters. However, the conventional chemical methods for the carboxylic acid reduction require strong reducing reagents such as metal hydrides, posting operational danger and low selectivity. In addition, the conversion of COOH into CHO requires particular hydride reagents, to avoid the further reduction to primary alcohols [26]. On the contrary, enzymatic reduction of carboxylic acids proceeds under mild reaction conditions with high selectivity and tolerance of other functional groups [27–28]. Currently, only a few members of this interesting type of enzymes (CAR, E.C.1.2.1.30) have been biochemically characterized (Table 1). They possess similar consensus sequence characteristics and reaction mechanism (post-translational phosphopantetheinylation, ATP, Mg^2+^, and NADPH as cofactors) [27–30]. As such, we initiated the search for new carboxylic acid reductases and the exploration of their potential as biocatalysts for the efficient bioreduction of carboxylic acids. Herein we report a new CAR from *Mycobacterium* sp. (*Mycobacterium* CAR) and its application in a chemo-enzymatic chain elongation method for the preparation of α,β-unsaturated esters by combining an enzymatic carboxylic acid reduction and Wittig reaction.

**Table 1:** Identified CARs (EC 1.2.1.30).

Identified CAR	Accession number	Origin	References

NiCAR^a,b^	AAR91681.1	*Nocardia iowensis*	[30–31]
MsCAR	WP_011855500.1	*Mycobacterium* sp. JLS	[32]
SgCAR	WP_012382217.1	*Streptomyces griseus* subsp. *griseus* NBRC 13350	[32]
MmCAR^a^	WP_012393886.1	*Mycobacterium marinum* M	[33]
SrCAR^a^	WP_013138593.1	*Segniliparus rotundus* DSM 44985	[28]
*Mycobacterium* CAR^a^	WP_019510583.1	*Mycobacterium* sp.	This work

^a^The protein was biochemically characterized and determined as a monomeric protein. ^b^The protein was also purified from natural strain.

## Results and Discussion

Nineteen actinomycete strains available in our laboratory were screened for the carboxylic acid reductase activity using phenylacetic acid (**1a**) as the substrate by GC analysis of the products. Among them, *Mycobacterium* sp. was found to catalyse the reduction of **1a** to 2-phenyl-1-ethanol (**1c**) with low conversion (Supporting Information File 1, Figure S1). The genome of *Mycobacterium* sp. strain was sequenced, and two gene sequences having 58% and 47% protein sequence identity with the NiCAR [30] (accession number AAR91681.1) were found by local BLAST search (tblastn) (Supporting Information File 1, Figure S2). These two genes were also found to have identical sequences with those (Gene ID 17912504 and Gene ID 17917114), respectively, in the genomic sequence of *Mycobacterium neoaurum* VKM Ac-1815D (accession no. CP006936) [34] by NCBI BLAST search (http://www.ncbi.nlm.nih.gov/BLAST/).

Since PPTase from *Mycobacterium* sp. has not been identified, a known *Nocardia* PPTase (accession number ABI83656.1) was selected for the post-translational phosphopantetheinylation in the current study [27–28]. The gene (Gene ID 17912504) was cloned into pET30b(+) and expressed in *E. coli*. The recombinant enzyme (*Mycobacterium* CAR) showed carboxylic acid reductase activity toward **1a**. The other gene (Gene ID 17917114) was cloned into pET28a(+) and expressed in *E. coli*. The gene was well expressed, but less fraction of soluble protein was obtained. The recombinant enzyme showed no carboxylic acid reductase activity toward **1a** when it was tested under same conditions as the gene 17912504 after having been treated with the PPTase enzyme.

The His-tagged *Mycobacterium* CAR and His-PPTase were produced as soluble protein as described in Supporting Information File 1 and purified in one chromatographic step each using a HisTrap^TM^ FF crude column (Supporting Information File 1, Figure S3). The molecular mass of His-CAR was estimated to be about 133 kDa by gel filtration chromatography. Since its theoretical value is 125 kDa, this enzyme is a monomeric protein. This is consistent with NiCAR [31] and SrCAR [28]. The carboxylic acid reductase activity was hardly detected when apo-CAR was used in the reaction system (**1a** as substrate) or the reaction system did not contain Mg^2+^ (Supporting Information File 1, Table S2), indicating that post-translational phosphopantetheinylation and Mg^2+^ were necessary for this enzymatic reduction, similar to the observations for the reaction with the carboxylic acid reductases from *Nocardia* [29,35] and *Segniliparus* [28]. The optimal pH and temperature for the enzymatic reduction of **1a** with *Mycobacterium* CAR were pH 9 and 25 °C, respectively. The apparent *K*_m_ and catalytic efficiencies (*k*_cat_/*K*_m_) of *Mycobacterium* CAR toward benzoic acid (**2a**) (Supporting Information File 1, Table S1) were 1.75 ± 0.16 mM and 0.93 mM^−1^·s^−1^, respectively. They were lower than those for NiCAR [31], SrCAR [28] and MnCAR [33].

In order to explore the application potential of *Mycobacterium* CAR, its substrate specificity was examined with the purified enzyme. The results in Table 2 showed that a series of aromatic and aliphatic carboxylic acids were reduced to their corresponding aldehydes. For the aliphatic acids, the reduction of nonanoic acid (**3a**) and lauric acid (**4a**) resulted in higher yields than those of hexanoic acid (**5a**) and pentadecanoic acid (**6a**), indicating that *Mycobacterium* CAR prefers the mid-chain fatty acids over the short-chain and long-chain ones. *Mycobacterium* CAR also showed activity toward different carboxylic acids with aromatic ring at the end carbon atom. For example, **1a** (75% yield), **2a** (94% yield), 3-phenylpropanoic acid (**7a**, 48% yield), and 4-phenylbutyric acid (**8a**) (64% yield) were reduced to the corresponding aldehydes. In contrast to *ortho*- and *meta*-methylbenzoic acid (**9a** and **10a**), *ortho*-hydroxylbenzoic acid (**11a**) was obviously the poorer substrate than *meta*-counterpart (**12a**), suggesting that in this case the activity of *Mycobacterium* CAR might be strongly influenced by the electronic properties other than the steric factor of the *ortho*-group on the benzene ring. This enzyme was less active toward 2-methylhexanoic acid (**13a**) and 2-phenylpropionic acid (**14a**) than **5a** and **1a**, respectively. This may be due to the sterically hindered effect of the α-methyl substitution, which is consistent with the results for *Pyrococcus furiosus* (whole-cell) [36] and NiCAR [31]. However, no significant difference was noted between 3-phenylbutyric acid (**15a**) and **7a**. *Mycobacterium* CAR showed good chemoselectivity for the reduction of some carboxylic acids containing C=C or C=O double bonds, such as linoleic acid (1**6a**), cinnamic acid (**17a**), ferulic acid (**18a**), and 3-benzoylpropionic acid (**19a**). These acids were reduced, with C=C or C=O double bonds remaining unaffected. However, this enzyme was less active toward **17a** than its saturated counterpart (**7a**). Interestingly, *Mycobacterium* CAR could effectively reduce ibuprofen (**20a**) more effectively than NiCAR and SrCAR, but it was not enantioselective. Rac-**13a**, rac-**14a** and rac-**15a** (5 mM) were almost completely transformed by *Mycobacterium* CAR (100 µg), suggesting it had no or low enantioselectivity toward these compounds.

**Table 2:** Substrate specificity of *Mycobacterium* CAR^a^.

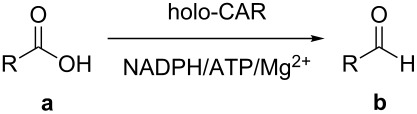		

Substrate		Analytic yield (%) of aldehyde

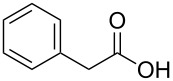	**1a**	75

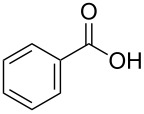	**2a**	94

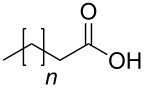	**3a** (*n* = 6)	100
	**4a** (*n* = 9)	100
	**5a** (*n* = 3)	68
	**6a** (*n* = 12)	3

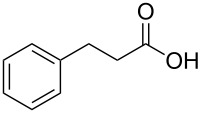	**7a**	48

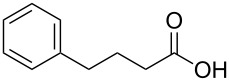	**8a**	64

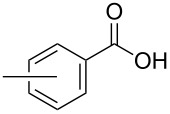	**9a** (*ortho*)	57
	**10a** (*meta*)	62

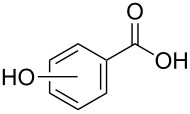	**11a** (*ortho*)	7
	**12a** (*meta*)	41

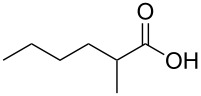	**13a**	62; 100^b^

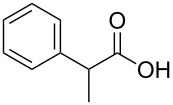	**14a** (*R*/*S*)	47; 100^b^

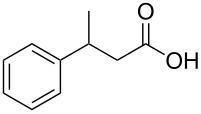	**15a**	44; 95^b^

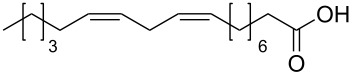	**16a**^c^	14

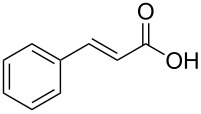	**17a**	36

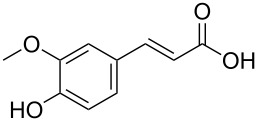	**18a**^d^	40

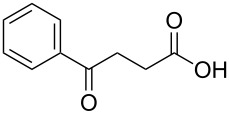	**19a**	68

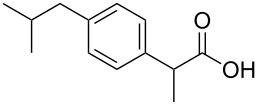	**20a** (*R*/*S*)	98

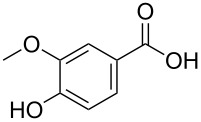	**21a**	76

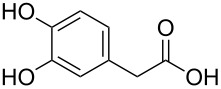	**22a**^c, d^	18

^a^Reaction conditions: Tris-HCl buffer (1 mL, 100 mM, pH 9) contained NADP^+^ (0.9 mM), GDH (1 U), glucose (60 mM), MgCl_2_ (10 mM), ATP (15 mM), substrate concentration (10 mM) and enzyme mixture (holo-CAR, 50 ug), 16 h, 25 °C, 200 rpm. ^b^Same as ^a^, but 5 mM of substrate and 100 μg of *Mycobacterium* CAR were used. ^c^Same as ^a^, but the reaction was performed in sodium phosphate buffer (100 mM, pH 7.5). ^d^Silylation was performed before the GC analysis.

The *Mycobacterium* CAR-catalysed carboxylic acid reduction was combined with Wittig reaction to establish a new chemo-enzymatic approach to the synthesis of α,β-unsaturated esters. As described in the Experimental section, the holo-CAR enzyme mixture was prepared and mixed with NADP^+^, GDH, glucose, ATP and carboxylic acid in Tris-HCl buffer. The reaction mixture was incubated at 25 °C for 16 h and extracted with ethyl acetate. The organic extract was concentrated to about 20 mL, and mixed with ethyl (triphenylphosphoranylidene)acetate and Na_2_CO_3_. After 24 h at room temperature, the organic solvent was removed under reduced pressure and the residue was purified by silica gel column chromatography to give the product (α,β-unsaturated ester). As shown in Table 3, α,β-unsaturated esters were obtained in moderate to high yields with *trans*-isomer as the major product. The corresponding α,β-unsaturated esters from aromatic carboxylic acids (**1a**, **2a**, **10a**, **12a** and **17a**) had higher yields than those from aliphatic ones (**3a**, **4a** and **5a**), and this might be due to lower yields of aliphatic aldehydes and higher loss in the product separation.

**Table 3:** Synthesis of α,β-unsaturated esters via enzymatic reduction and Wittig reaction^a^.

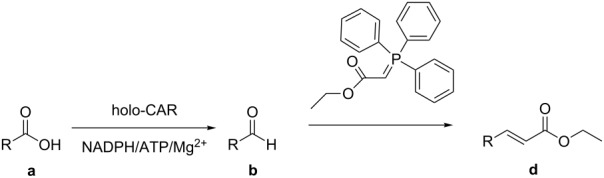			

Substrate (**a**)	Analytic yield (%) of aldehydes (**b**)^b^	Isolated yield (%) of α,β-unsaturated esters (**d**)	Ratio *E*/*Z*^c^

**1a**	72	60	68:32
**2a**	100	70	92:8
**3a**	65	41	87:13
**4a**	70	46	89:11
**5a**	67	38	96:4
**10a**	100	81	92:8
**12a**	79	59	90:10
**17a**	78	65	94:6

^a^The carboxylic acid was first reduced to aldehyde, after being extracted with ethyl acetate, ethyl (triphenylphosphoranylidene)acetate was added for the Wittig reaction. ^b^Determined by GC analysis of the reaction mixture. ^c^Determined by GC analysis of isolated products.

## Conclusion

A new CAR from *Mycobacterium* sp. was successfully cloned, overexpressed and identified. It exhibited a broad substrate spectrum and was active toward both aliphatic and aromatic carboxylic acids, including ibuprofen. Other functional groups such as keto groups and C=C double bonds remained unaffected. *Mycobacterium* CAR catalysed carboxylic acid reduction to give aldehydes, followed by a Wittig reaction to afford α,β-unsaturated esters with extension of two carbon atoms. This study demonstrates a new chemo-enzymatic chain elongation method for the synthesis of these important compounds from bio-based fatty and aromatic acids of natural resources. However, the enzymatic reduction of carboxylic acids requires CoA, ATP and NADPH, and this still presents challenge for its application at large scale, which may be overcome by using the whole cell catalyst of the engineered enzyme production strain with efficient amount of CoA, ATP and NADPH or effective regeneration systems of them.

## Experimental

### Cloning of *Mycobacterium* CAR gene

*Mycobacterium* sp. chromosomal DNA (gDNA) was extracted and puriﬁed using a TIANamp Bacteria DNA Kit. The *Mycobacterium* CAR gene (Gene ID 17912504) was amplified by PCR using *Mycobacterium* gDNA as template and primers containing the restriction sites *Nde*I and *Xho*I, respectively, CAR-F 5′-CATGCATATGTTCGCCGAAAATCTTGATGACCAG-3′ and CAR-R 5′-CATCTCGA GCAGCAGGCCGAGCAATTGCAGGT-3′. The PCR fragment was puriﬁed and then ligated with cloning vector pJET1.2/blunt, which was confirmed by DNA sequencing. A CAR DNA fragment was acquired from the vector pJET1.2-CAR by digesting at the restriction sites *Nde*I and *Xho*I, and then ligated by T4 DNA ligase into pET30b(+) at the same restriction sites to generate the expression vector pET30b(+)-CAR. The confirmed recombinant vector was transformed into *E. coli* BL21(DE3).

### Expression and purification of *Mycobacterium* CAR and *Nocardia* PPTase

A culture of *E. coli* BL21 (DE3) cells harboring pET30b(+)-CAR or pET32a(+)-PPTase was grown overnight in LB-ampicillin (100 µg/mL) medium (5 mL) at 37 °C, and then inoculated into 1 L of LB-ampicillin (100 µg/mL) medium. The resulting culture was incubated continually at 200 rpm in a rotary shaker at 37 °C until cells reached mid-log growth (OD_600_ of 0.5–1.0), which was followed by the addition of 0.5 mM IPTG and further incubation for 12 h at 25 °C. Cells were harvested by centrifugation at 12 000*g* for 10 min at 4 °C, and disrupted by high pressure homogenizer after re-suspension in binding buffer (20 mM sodium phosphate buffer, 0.5 M NaCl, 20 mM imidazole, pH 7.4). His-CAR or His-PPTase protein in the supernatant fraction was collected from the crude cell lysate by centrifugation at 12 000*g* for 20 min. Protein purification was performed on a HisTrap^TM^ FF crude column (GE Healthcare, Piscataway, USA), and the protein was desorbed with an elution buffer (20 mM sodium phosphate, 0.5 M NaCl, 0.5 M imidazole, pH 7.4). The purified proteins His-CAR or His-PPTase were dialyzed in a sodium phosphate buffer (50 mM, pH 7.5) and then stored at −20 °C for further use.

### Standard reduction procedure

The His-CAR (1.3 mg) was incubated with His-PPTase (256 µg) in the presence of CoA (1 mM) as a cofactor for 1 h at 28 °C in a final volume of 520 µL of sodium phosphate buffer (100 mM, pH 7.5) containing 10 mM of MgCl_2_. The resulting enzyme mixture (holo-CAR, 50 or 100 µg) was mixed with NADP^+^ (0.9 mM), GDH (1 U, one unit corresponds to the amout of enzyme which could convert 1 µmol NADP^+^ to NADPH per minute using D-glucose as the substrate), glucose (60 mM), MgCl_2_ (10 mM), carboxylic acid (5 or 10 mM, from 1 M stock solution in DMSO), and ATP (15 mM) in Tris-HCl buffer (100 mM, pH 9) with a final volume of 1 mL. The reaction mixture was incubated at 200 rpm in a rotary shaker at 25 °C for 16 h, and extracted with 1 mL of ethyl acetate after the pH was adjusted to 2–3 with 1 M HCl solution. The organic extracts were dried over anhydrous sodium sulfate and analysed by gas chromatography (GC) to determine the amount of substrate (**a**) and products (aldehyde **b**) in the mixture. All experiments were conducted in triplicate.

### Substrate specificity

The reduction of a series of carboxylic acids was carried out by following the standard reduction procedure. The yields were determined by GC analysis.

### Experimental procedures for the synthesis of compounds **1d**, **2d**, **3d**, **4d**, **5d**, **10d**, **12d** and **17d**

A typical procedure was as follows using ethyl 4-phenylbut-2-enoate (**1d**) as the example. The enzyme mixture (holo-CAR, 0.5 mg/mL) was prepared as described above, and was mixed with NADP^+^ (0.45 mM), GDH (1 U), glucose (60 mM), ATP (13 mM) and phenylacetic acid (**1a**, 10 mM) in Tris-HCl buffer (total volume 25 mL, 100 mM, pH 9). The reaction mixture was incubated at 100 rpm in a rotary shaker at 25 °C for 16 h, and extracted 3 times with 25 mL of ethyl acetate. The organic extract was concentrated to about 20 mL under reduced pressure, and then ethyl (triphenylphosphoranylidene)acetate (100 mM) and Na_2_CO_3_ (about 0.5 g) were added, and the reaction mixture was stirred for 24 h at room temperature. The organic solvent was removed under reduced pressure and the residue was purified by silica gel column chromatography to give the product, **1d** (30.1 mg, 60%) was obtained.

## Supporting Information

File 1 Materials, bacterial screening, analytical procedures, NMR data and spectra of **1d**, **2d**, **3d**, **4d**, **5d**, **10d**, **12d** and **17d**.
