# Supplementary material for: Synthesis of α,β-unsaturated esters via a chemo-enzymatic chain elongation approach by combining carboxylic acid reduction and Wittig reaction
Source: Beilstein J Org Chem. 2015 Nov 19;11:2245–51. doi: 10.3762/bjoc.11.243 (PMC4661009; doi:10.3762/bjoc.11.243)
Supplement: File 1 — Materials, bacterial screening, analytical procedures, NMR data and spectra of 1d, 2d, 3d, 4d, 5d, 10d, 12d and 17d. [file Beilstein_J_Org_Chem-11-2245-s001.pdf]

**Supporting Information**

**for**

# Synthesis of α,β-unsaturated esters via a chemo-enzymatic chain elongation approach by combining carboxylic acid reduction and Wittig reaction

Yitao Duan, Peiyuan Yao, Yuncheng Du, Jinhui Feng, Qiaqing Wu* and Dunming Zhu*

Address: National Engineering Laboratory for Industrial Enzymes and Tianjin Engineering Center for Biocatalytic Technology, Tianjin Institute of Industrial Biotechnology, Chinese Academy of Sciences, Tianjin 300308, P. R. China

Email: Qiaqing Wu* - wu_qq@tib.cas.cn; Dunming Zhu* - zhu_dm@tib.cas.cn

*Corresponding author

**Materials, bacterial screening, analytical procedures, NMR data and spectra of 1d, 2d, 3d, 4d, 5d, 10d, 12d and 17d.**

## Materials

Nineteen actinomycete strains were isolated from soil samples and stored in our laboratory. Nutrient-rich medium (NRM, pH 7.0) contained 15.0 g of glucose, 5.0 g of peptone, 5.0 g of yeast extract, 1.0 g of $KH_2PO_4$, 1.0 g of $K_2HPO_4$, 1.0 g of NaCl and

0.5 g of $MgSO_4 \cdot 7H_2O$ in 1 L of distilled water. Phusion DNA Polymerase, T4 DNA Ligase, CloneJET™ PCR Cloning Kit and FastDigest Restriction Enzymes were purchased from Fermentas (Shenzhen, China). A pEASY®-Uni Seamless Cloning and Assembly Kit was purchased from TransGen Biotech (Beijing, China). Vectors pET30b(+) and pET28a(+) were purchased from Novagen (Schwalbach, Germany). The plasmid extraction kit was from CWBIO (Beijing, China). The TIANamp Bacteria DNA Kit and gel extraction kit were from Tiangen Biotech (Beijing, China). The lyophilised powder of glucose dehydrogenase (GDH) from *Bacillus subtilis,* with a specific activity of 1.1 U/mg, was prepared in our laboratory. Triphenylphosphorane was purchased from Alligator Reagent (Nanjing, China). All carboxylic acids were purchased from Sinopharm Chemical Reagent Co. Ltd (Shanghai, China), Alfa Aesar (Shanghai, China) or Sigma-Aldrich (St. Louis, USA). $^{1}H$ and $^{13}C$ NMR spectra were recorded on a Bruker AVANCE-III 400 MHz NMR spectrometer (Bruker BioSpin, Rheinstetten, Germany).

## Bacterial screening

Each of the nineteen actinomycete strains was inoculated into 5 mL of sterilized NRM for 20 h and 2 mL of the culture liquid (inoculum size 1:50) was transferred into 100 mL of NRM, which was constantly shaken at 30 °C and 200 rpm for 24 h. The cells were collected by centrifugation (6,000 rpm, 15 min) and were re-suspended in 0.1 M potassium phosphate buffer (pH 7.0). Phenylacetic acid (**1a**) (10 mM) and glucose (4 mg) were added into a suspension of resting cells (0.2–0.3 g, wet weight) in 1 mL of potassium phosphate buffer (0.1 M, pH 7.5). The reaction mixture was incubated at 200 rpm in a rotary shaker at 30 °C for 24 h, and extracted with 1 mL of ethyl acetate after the pH was adjusted to 2–3 with 1 M HCl solution. The organic extracts were dried over anhydrous sodium sulfate and analyzed by gas chromatography (GC) to

determine the amount of substrate (**a**) and products (aldehyde **b**, alcohol **c**) in the mixture. One of the 19 actinomycetes catalysed the reduction of phenylacetic acid (**1a**) to give phenethyl alcohol (**1c**) in small amount (Figure S1), and the strain was noted as *Mycobacterium* sp.

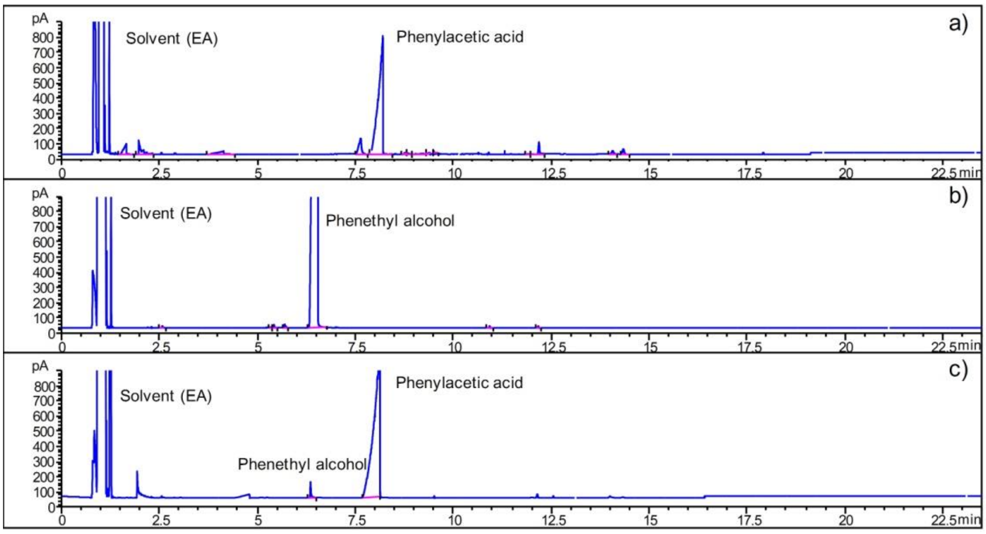


**Figure S1:** GC analysis of the reduction of phenylacetic acid (**1a**) catalyzed by *Mycobacterium* sp. a) **1a**. b) Phenethyl alcohol (**1c**). c) The reduction of **1a** catalyzed by *Mycobacterium* sp.

## Genome analysis of *Mycobacterium* sp.

The genomic sequence of *Mycobacterium* sp. was sequenced by our group (data has not yet been released). Two highly match sequences with the *Nocardia* CAR (accession number AAR91681.1) [1] were found by local BLAST search (tblastn) in the *Mycobacterium* sp. genome. NCBI BLAST search (http://www.ncbi.nlm.nih.gov/BLAST/) using these two sequences of *Mycobacterium* sp. genome as template, respectively, showed that two genes (Gene ID 17912504 and Gene ID 17917114) with identical sequences existed in the *Mycobacterium neoaurum* VKM Ac-1815D genome [2]. The sequence analysis of two putative carboxylic acid reductase with NiCAR is shown in Figure S2.

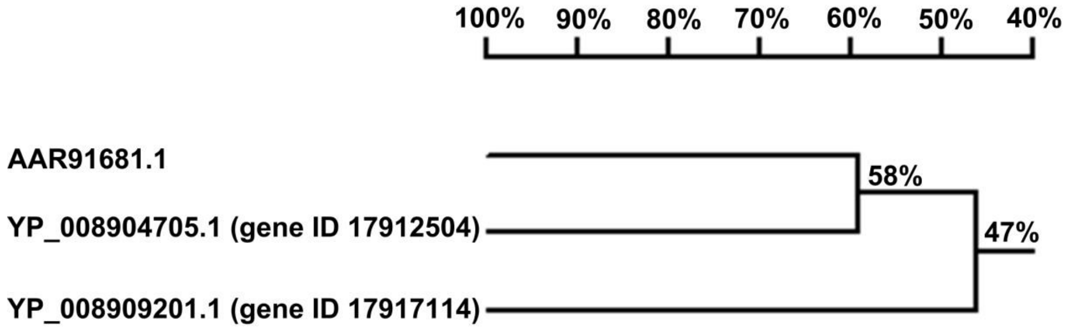


**Figure S2:** The sequence analysis of two putative carboxylic acid reductase with NiCAR.

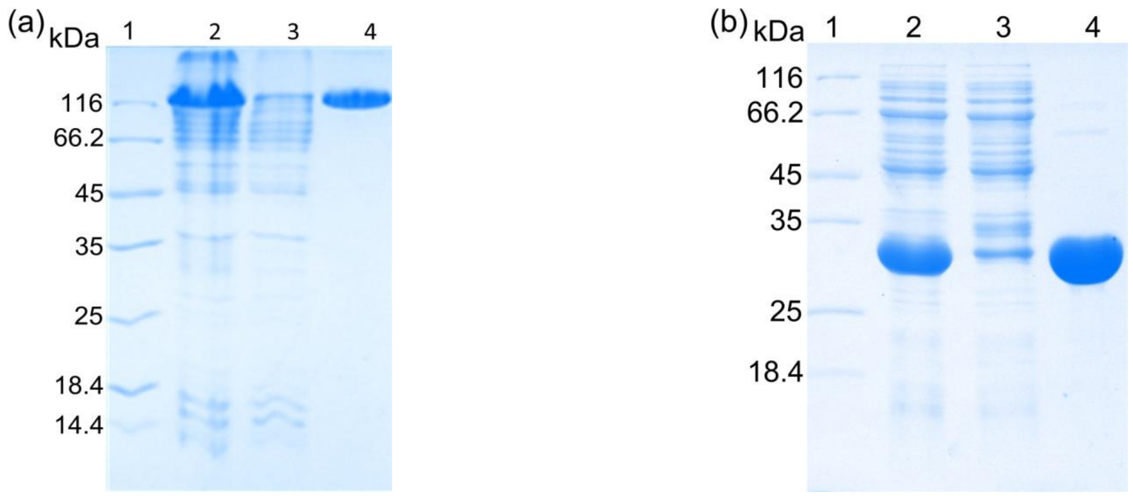


**Figure S3:** Purification of recombinant proteins His-CAR and His-PPTase by affinity chromatography. (a) Lane 1: Protein marker; Lane 2: Lysate supernatant of *E. coli* BL21(DE3) harboring pET30b(+)-CAR; Lane 3: Eluting fraction before desorption of CAR; Lane 4: Purified His-CAR protein. (b) Lane 1: Protein marker; Lane 2: Lysate supernatant of *E. coli* BL21(DE3) harboring pET32a(+)-PPTase; Lane 3: Eluting fraction before desorption of PPTase; Lane 4: Purified His-PPTase protein.

## Molecular weight of *Mycobacterium* CAR

The molecular mass of *Mycobacterium* CAR was determined by gel filtration chromatography using a Superdex 200 10/300 GL column and the

high-molecular-weight gel filtration calibration kit (GE Healthcare, Piscataway, USA) was used for calibration. The sodium phosphate buffer (50 mM, pH 7) containing 150 mM NaCl was used as the eluent. The flow rate was 0.4 mL/min and the absorbance at 280 nm was monitored.

## Determination of kinetic parameters

The enzyme mixtures were prepared by following the standard reduction procedure. The reaction was performed in Tris-HCl buffer (100 mM, pH 9) containing enzyme mixture (holo-CAR, 20 µL, 12.5 µg), $MgCl_2$ (10 mM), varying concentrations of benzoic acid (**2a**, 0.01–12 mM), ATP (1.2 mM), and NADPH (0.4 mM) in a final volume of 200 µL. For ATP, **2a** (4 mM), NADPH (0.4 mM), enzyme mixture (holo-CAR, 20 µL, 12.5 µg) and ATP in the range of 0.025–2 mM were used for the activity assay. For NADPH, **2a** (4 mM), ATP (1.2 mM), enzyme mixture (holo-CAR, 20 µL, 12.5 µg) and NADPH in the range of 0.005–0.5 mM were used for the activity assay. The activity of CAR was determined by spectrophotometrically measuring the oxidation of NADPH at 340 nm ($\varepsilon$ = 6.22 $mM^{-1}\cdot cm^{-1}$). The specific activity was defined as the number of µmol of NADPH converted in one minute by 1 mg of enzyme ($\mu mol\cdot min^{-1}\cdot mg^{-1}$). All experiments were conducted in triplicate. The kinetic parameters were obtained by measuring the initial velocities of the enzymatic reaction and curve-fitting according to the Michaelis–Menten equation using GraphPad Prism 5 software (GraphPad Software Inc., San Diego, USA).

**Table S1:** The apparent kinetic properties of *Mycobacterium* CAR.

| Substrate | *Mycobacterium* CAR | |
|---|---|---|
| | $K_m$ (mM) | $k_{cat}$ ($s^{-1}$) |
| Benzoic acid | 1.75 ± 0.16 | 1.62 ± 0.08 |
| ATP | 0.29 ± 0.02 | |
| NADPH | 0.04 ± 0.01 | |

## Effects of post-translational phosphopantetheinylation and $Mg^{2+}$ in *Mycobacterium* CAR-catalysed reduction

For the effect of $Mg^{2+}$ and post-translational phosphopantetheinylation, the following reaction mixtures of sodium phosphate buffer (1 mL, 100 mM, pH = 7.5) contained His-CAR (apo-CAR) or enzyme mixture (holo-CAR) (50 µg), **1a** (10 mM, from 1 M stock solution in DMSO), $NADP^+$ (0.9 mM), GDH (1 U), glucose (60 mM), ATP (15 mM) and $MgCl_2$ (0 mM or 10 mM) (Table S2). The reaction mixtures were incubated at 200 rpm in a rotary shaker at 30 °C for 12 h. And yield of phenylacetaldehyde (**1b**) was expressed as a molar ratio of product (**1b**) to product plus substrate (**1a**) determined by GC.

**Table S2:** Effects of post-translational phosphopantetheinylation and $Mg^{2+}$ on enzymatic reaction.

| Conditions | Yield (%) |
|---|---|
| | 30 °C |
| Enzyme mixtures (holo-CAR) + $MgCl_2$ | 23 |
| His-CAR (apo-CAR) + $MgCl_2$ | 2 |
| Enzyme mixtures (holo-CAR) | 1.1 |

## Analytical procedures

GC was performed using a HP-5 capillary column (30 m × 0.25 mm × 0.25 µm) with flame ionization detector. The carrier gas was helium at a flow rate of 2 mL/min. The column temperature was controlled as follows: 60 °C (4 min) — 20 °C/min — 200 °C (6 min) — 20 °C/min — 250 °C (4 min). The identities of these products were

confirmed with authentic samples synthesized by enzymatic methods [3]. Yield was expressed as a molar ratio of product to product plus substrate determined by GC.

## NMR data and spectra of 1d, 2d, 3d, 4d, 5d, 10d, 12d and 17d

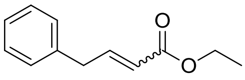

**1d**

**Ethyl 4-phenylbut-2-enoate (1d). 1d** (30.1 mg, 60%) was obtained from **1a** (36 mg). $^1$H NMR (400 MHz, $CDCl_3$) δ 7.19 - 7.27 (m, 2 H), 7.13 - 7.19 (m, 2 H), 7.10 (d, $J$ = 7.3 Hz, 1 H), 7.02 (dt, $J$ = 15.6, 6.9 Hz, 0.7 H, *trans*), 6.21 - 6.32 (m, 0.3 H, *cis*), 5.68-5.81 (m, 1 H), 4.06 - 4.18 (m, 2 H), 3.95 (d, $J$ = 7.3 Hz, 0.6 H, *cis*), 3.44 (d, $J$ = 6.6 Hz, 1.4 H, *trans*), 1.14 - 1.27 (m, 3 H). $^{13}$C NMR (100 MHz, $CDCl_3$) δ 166.48, 166.40, 147.92, 147.25, 139.51, 137.74, 128.83, 128.70, 128.63, 126.68, 126.34, 122.41, 119.97, 60.28, 60.02, 38.48, 35.15, 14.30, 14.26.

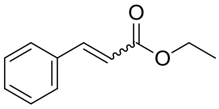

**2d**

**Ethyl cinnamate (2d).** By following the same procedure described for **1d**, **2d** (37 mg, 70%) was obtained from benzoic acid (**2a**) (36.6 mg). $^1$H NMR (400 MHz, $CDCl_3$) δ 7.62 (d, $J$ = 16.1 Hz, 0.92 H, *trans*), 7.40 - 7.54 (m, 2 H), 7.23 - 7.37 (m, 3 H), 6.88 (d, $J$ = 12.5 Hz, 0.08 H, *cis*), 6.37 (d, $J$ = 16.1 Hz, 0.92 H, *trans*), 5.88 (d, $J$ = 12.5 Hz, 0.08 H, *cis*), 4.20 (q, $J$ = 7.1 Hz, 1.84 H, *trans*), 4.11 (q, $J$ = 7.1 Hz, 0.16 H, *cis*), 1.27 (t, $J$ = 7.1 Hz, 3 H). $^{13}$C NMR (100 MHz, $CDCl_3$) δ 167.00, 144.59 (*trans*), 142.98 (*cis*), 134.50, 130.22 (*trans*), 129.70 (*cis*), 128.98 (*cis*), 128.89 (*trans*), 128.06 (*trans*), 127.99 (*cis*), 119.92 (*cis*), 118.31 (*trans*), 60.50, 14.34.

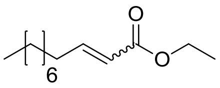

**3d**

**Ethyl undec-2-enoate (3d).** By following the same procedure described for **1d**, **3d** (20 mg, 41%) was obtained from nonanoic acid (**3a**) (36.3 mg). $^1$H NMR (400 MHz, $CDCl_3$): δ 6.96 (dt, *J* = 15.7, 7.0 Hz, 1 H), 5.81 (d, *J* = 15.7 Hz, 1 H), 4.18 (q, *J* = 7.1 Hz, 2 H), 2.15 - 2.23 (m, 2 H), 1.39 - 1.50 (m, 2 H), 1.20 - 1.36 (m, 13 H), 0.88 (t, *J* = 6.7 Hz, 3 H). $^{13}$C NMR (100 MHz, $CDCl_3$): δ 166.82, 149.52, 121.23, 60.12, 32.22, 31.86, 29.37, 29.20, 29.17, 28.04, 22.67, 14.31, 14.10.

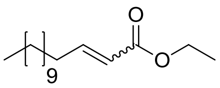

**4d**

**Ethyl tetradec-2-enoate (4d).** By following the same procedure described for **1d**, **4d** (23 mg, 46%) was obtained from lauric acid (**4a**) (39.7 mg). $^1$H NMR (400 MHz, $CDCl_3$): δ 6.96 (dt, *J* = 15.5, 6.9 Hz, 1 H), 5.81 (d, *J* = 15.7 Hz, 1 H), 4.18 (q, *J* = 7.1 Hz, 2 H), 2.15 - 2.23 (m, 2 H), 1.39 - 1.50 (m, 2 H), 1.20 - 1.36 (m, 19 H), 0.88 (t, *J* = 6.7 Hz, 3 H). $^{13}$C NMR (100 MHz, $CDCl_3$): δ 166.81, 149.51, 121.23, 60.12, 32.22, 31.93, 29.65, 29.63, 29.54, 29.41, 29.35, 29.17, 28.05, 22.71, 14.30, 14.12.

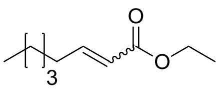

**5d**

**Ethyl oct-2-enoate (5d).** By following the same procedure described for **1d**, **5d** (20.2 mg, 38%) was obtained from hexanoic acid (**5a**) (36.2 mg). $^1$H NMR (400 MHz, $CDCl_3$) δ 6.90 (dt, *J* = 15.6, 7.0 Hz, 0.9 H, *trans*), 6.15 (dt, *J* = 11.5, 7.6 Hz, 0.1 H, *cis*), 5.74 (d, *J* = 15.6 Hz, 0.9 H, *trans*), 5.68 (d, *J* = 11.5 Hz, 0.1 H, *cis*), 4.08 - 4.16 (m, 2 H), 2.57 (qd, *J* = 7.5, 1.5 Hz, 0.2 H, *cis*), 2.07 - 2.17 (m, 1.8 H, *trans*), 1.33 - 1.44 (m, 2 H), 1.16 - 1.29 (m, 7 H), 0.82 (t, *J* = 6.8 Hz, 3 H). $^{13}$C NMR (100 MHz, $CDCl_3$) δ 166.80, 149.48, 121.23, 60.11, 32.16, 31.31, 27.70, 22.43, 14.29, 13.95.

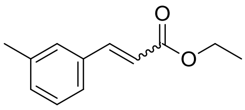


**10d**

**Ethyl 3-(*m*-tolyl)acrylate (10d).** By following the same procedure described for **1d**, **10d** (38.6 mg, 81%) was obtained from 3-methylbenzoic acid (**10a**) (33.9 mg). $^1$H NMR (400 MHz, $CDCl_3$) δ 7.60 (d, *J* = 16.1 Hz, 0.92 H, *trans*), 7.24 - 7.34 (m, 2 H), 7.16 - 7.24 (m, 1 H), 7.04 - 7.16 (m, 1 H), 6.84 (d, *J* = 12.7 Hz, 0.08 H, *cis*), 6.31-6.40 (d, *J* = 16.1 Hz, 0.92 H, *trans*), 5.86 (d, *J* = 12.7 Hz, 0.08 H, *cis*), 4.20 (q, *J* = 7.3 Hz, 1.84 H, *trans*), 4.11 (q, *J* = 7.1 Hz, 0.16 H, *cis*), 2.27-2.33 (m, 3 H), 1.27 (t, *J* = 7.1 Hz, 3 H). $^{13}$C NMR (100 MHz, $CDCl_3$) δ 167.07, 144.76 (*trans*), 142.96 (*cis*), 138.53 (*trans*), 137.52 (*cis*), 134.86 (*cis*), 134.45 (*trans*), 131.06 (*trans*), 130.32 (*cis*), 129.75 (*cis*), 128.77 (*trans*), 128.73 (*trans*), 127.91 (*cis*), 126.79 (*cis*), 125.24 (*trans*), 119.75 (*cis*), 118.08 (*trans*), 60.45 (*trans*), 60.26 (*cis*), 21.37 (*cis*), 21.32 (*trans*), 14.34 (*trans*), 14.10 (*cis*).

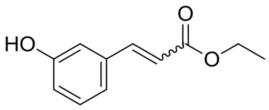


**12d**

**Ethyl 3-(3-hydroxyphenyl)acrylate (12d).** By following the same procedure described for **1d**, **12d** (28 mg, 59%) was obtained from 3-hydroxybenzoic acid (**12a**) (34.3 mg). $^1$H NMR (400 MHz, $CDCl_3$) δ 7.64 (d, *J* = 16.1 Hz, 1 H), 7.21 - 7.28 (m, 1 H), 7.20 (s, 1 H), 7.08 (d, *J* = 7.6 Hz, 1 H), 7.03 (s, 1 H), 6.89 (dd, *J* = 8.1, 2.2 Hz, 1 H), 6.40 (d, *J* = 15.9 Hz, 1 H), 6.21 (d, *J* = 5.1 Hz, 1 H), 5.95 (d, *J* = 12.5 Hz, 0.1 H, *cis*), 4.28 (q, *J* = 7.3 Hz, 2 H), 1.34 (t, *J* = 7.1 Hz, 3 H). $^{13}$C NMR (100 MHz, $CDCl_3$) δ 167.63, 156.34, 144.90, 135.83, 130.12, 120.63, 118.32, 117.85, 114.68, 60.90, 14.28.

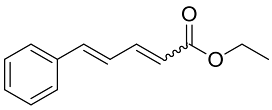

**17d**

**Ethyl 5-phenylpenta-2,4-dienoate (17d).** By following the same procedure described for **1d**, **17d** (33 mg, 65%) was obtained from *trans*-cinnamic acid (**17a**) (37.4 mg). $^{1}$H NMR (400 MHz, $CDCl_3$) δ 7.40 - 7.49 (m, 3 H), 7.28 - 7.40 (m, 3 H), 6.86 - 6.91 (m, 2 H), 5.99 (d, *J* = 15.4 Hz, 1 H), 4.20 - 4.27 (m, 2 H), 1.32 (t, *J* = 7.1 Hz, 3 H). $^{13}$C NMR (100 MHz, $CDCl_3$) δ 167.07, 144.53, 140.36, 136.08, 129.03, 128.82, 127.20, 126.28, 121.37, 60.36, 14.34.

## NMR Spectra of 1d, 2d, 3d, 4d, 5d, 10d, 12d and 17d

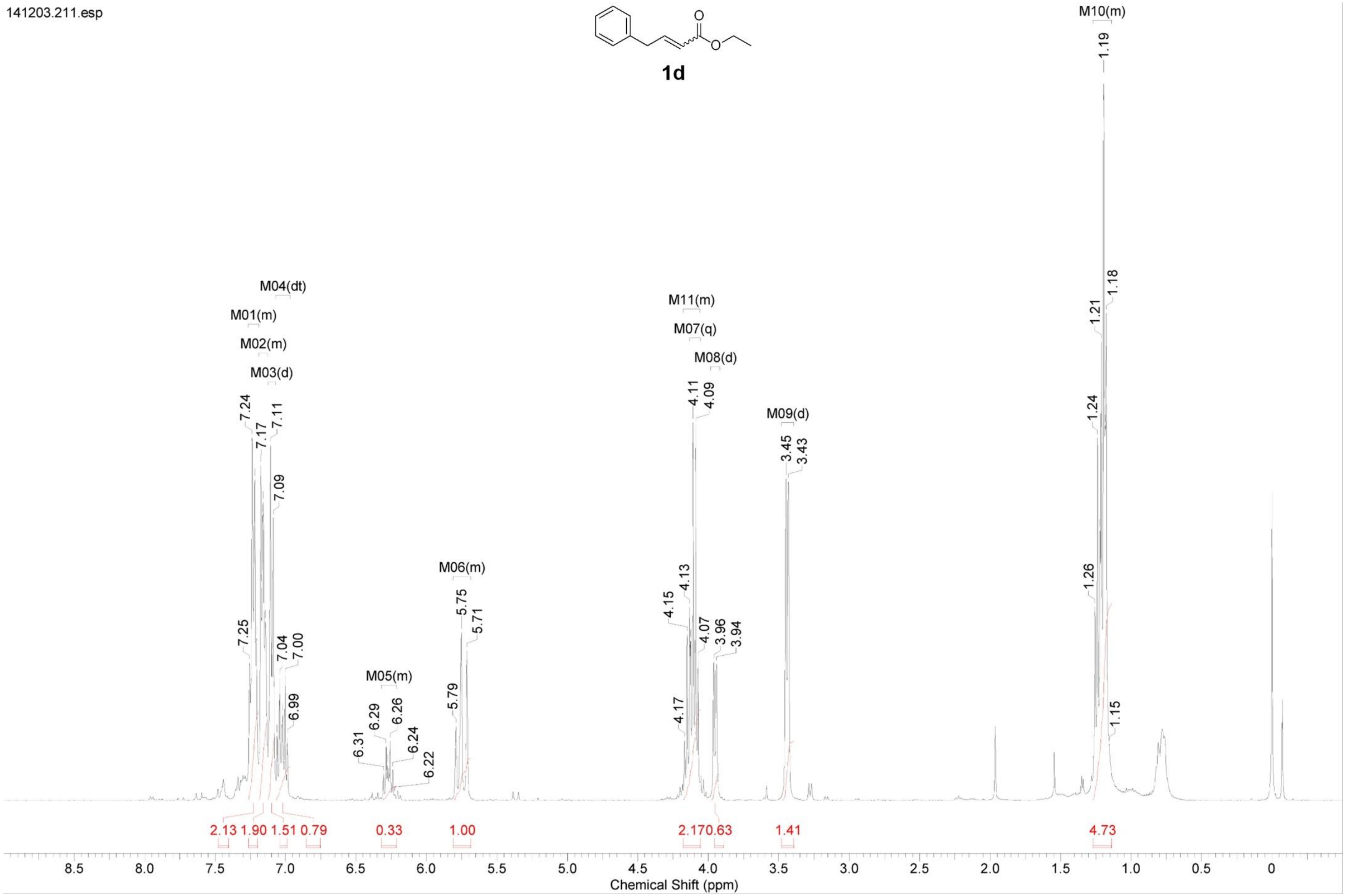
141203.211.esp
1d
M10(m)
1.19
1.18
1.21
1.24
1.26
1.15
M04(dt)
M01(m)
M02(m)
M03(d)
7.24
7.17
7.11
7.09
7.25
7.04
7.00
6.99
M05(m)
6.31
6.29
6.26
6.24
6.22
M06(m)
5.75
5.71
5.79
M11(m)
M07(q)
M08(d)
4.11
4.09
4.13
4.15
4.07
3.96
3.94
4.17
M09(d)
3.45
3.43
2.13
1.90
1.51
0.79
0.33
1.00
2.17
0.63
1.41
4.73
8.5
8.0
7.5
7.0
6.5
6.0
5.5
5.0
4.5
4.0
3.5
3.0
2.5
2.0
1.5
1.0
0.5
0
Chemical Shift (ppm)

141203.212.esp

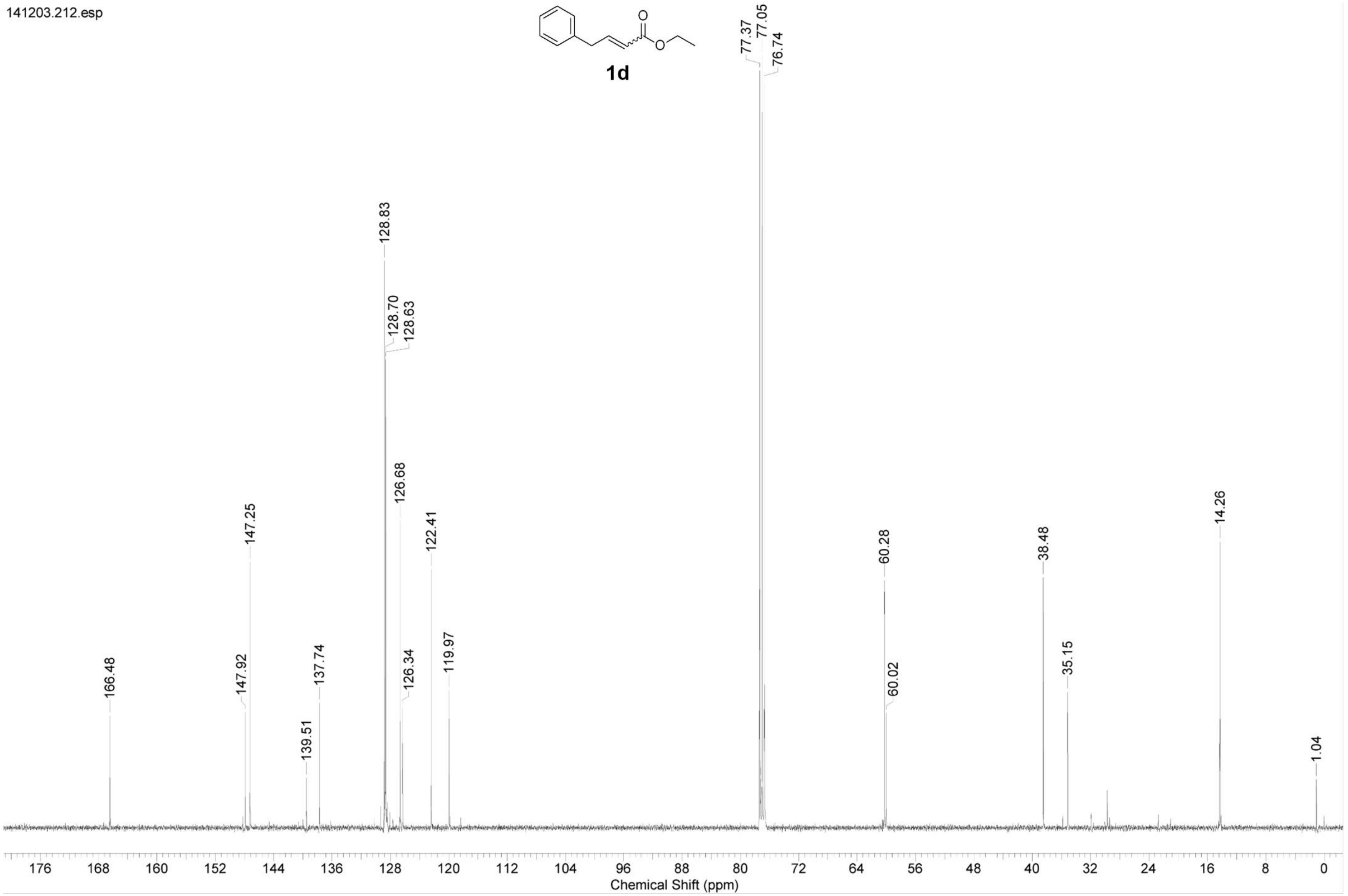

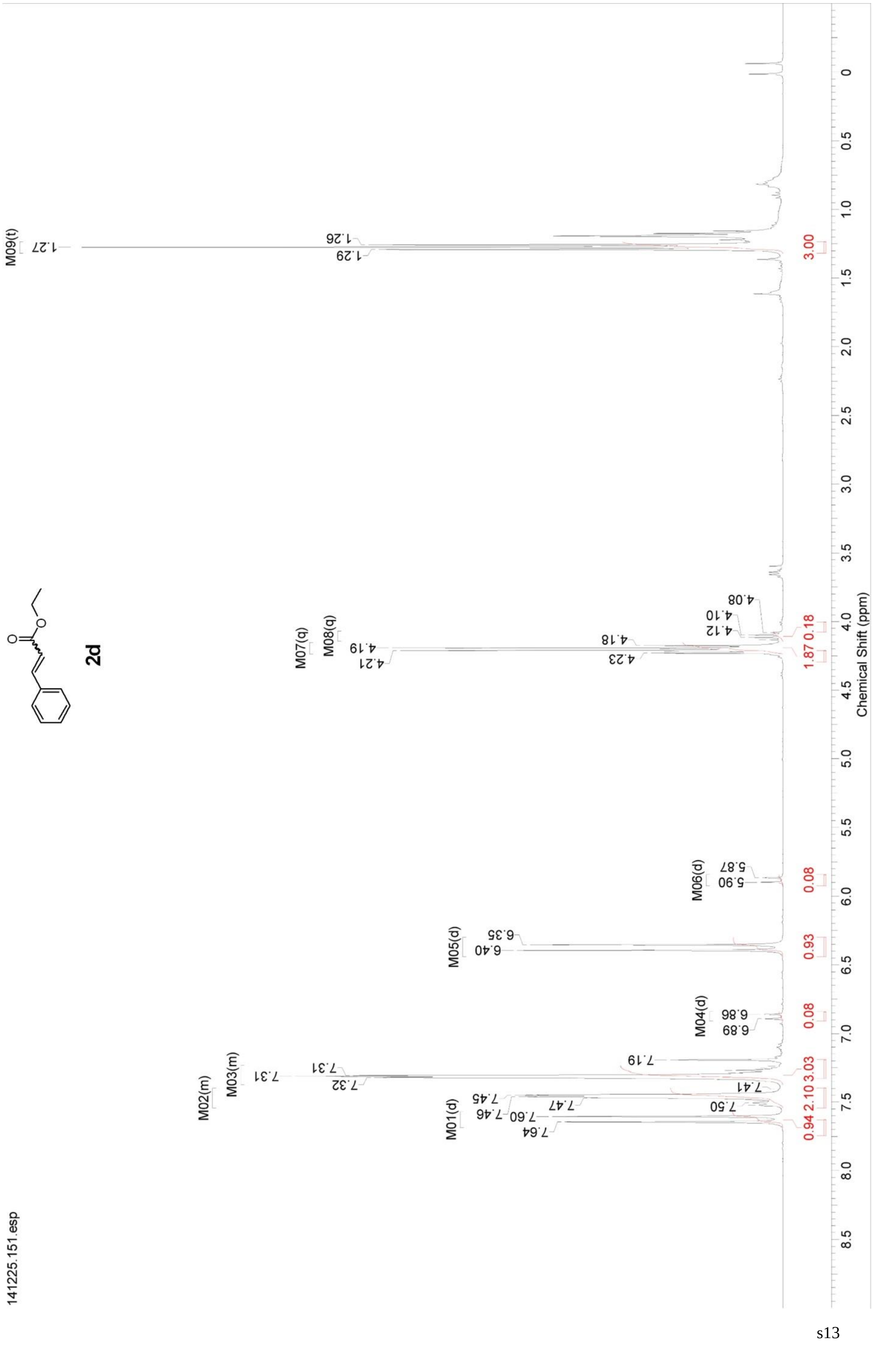
141225.151.esp
2d
M01(d)
M02(m)
M03(m)
M04(d)
M05(d)
M06(d)
M07(q)
M08(q)
M09(t)
7.64
7.60
7.50
7.47
7.46
7.45
7.41
7.32
7.31
7.19
6.89
6.86
6.40
6.35
5.90
5.87
4.23
4.21
4.19
4.18
4.12
4.10
4.08
1.29
1.27
1.26
0.94
2.10
3.03
0.08
0.93
0.08
1.87
0.18
3.00
Chemical Shift (ppm)

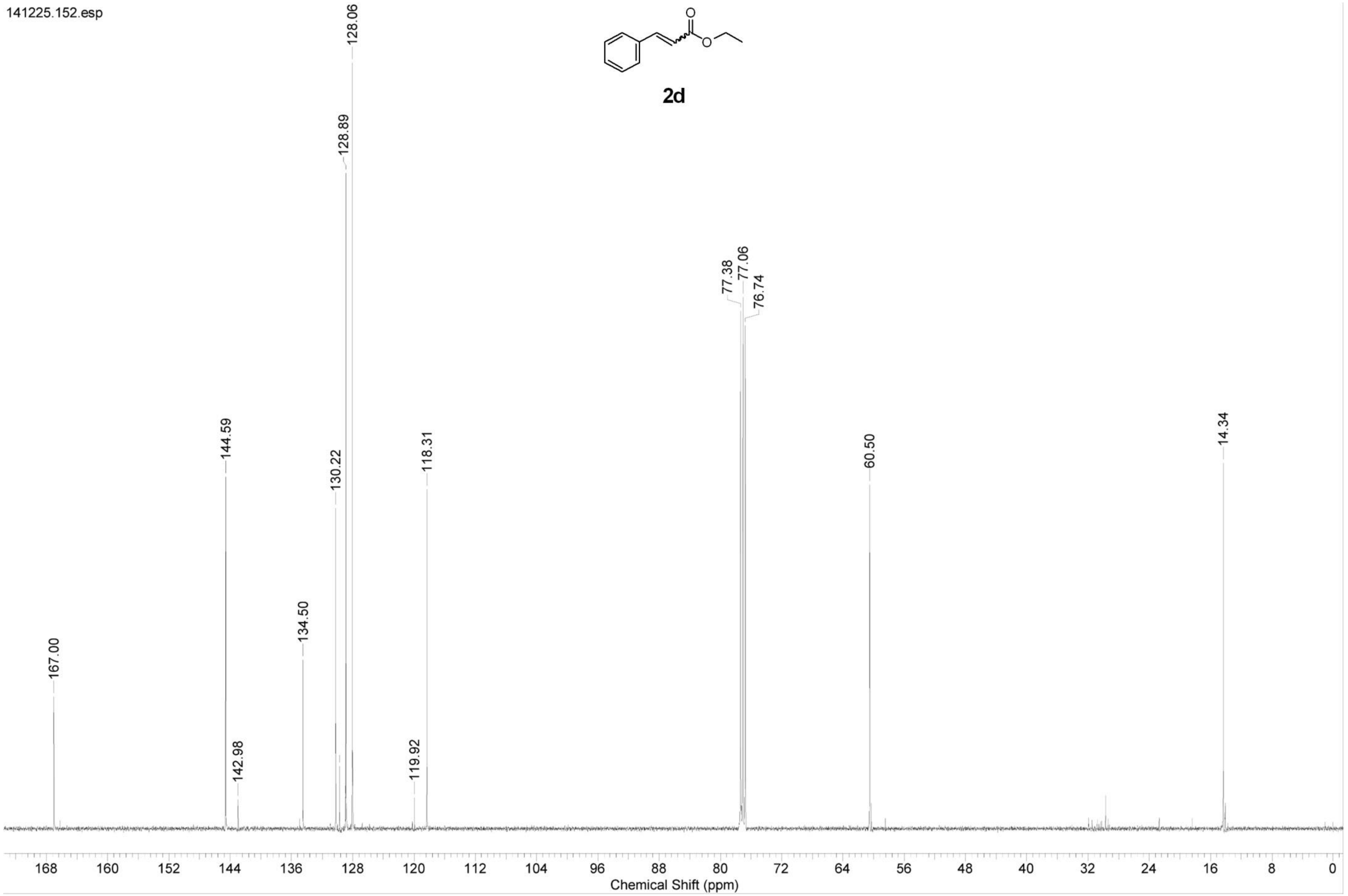
141225.152.esp
2d
167.00
144.59
142.98
134.50
130.22
128.89
128.06
119.92
118.31
77.38
77.06
76.74
60.50
14.34
168 160 152 144 136 128 120 112 104 96 88 80 72 64 56 48 40 32 24 16 8 0
Chemical Shift (ppm)

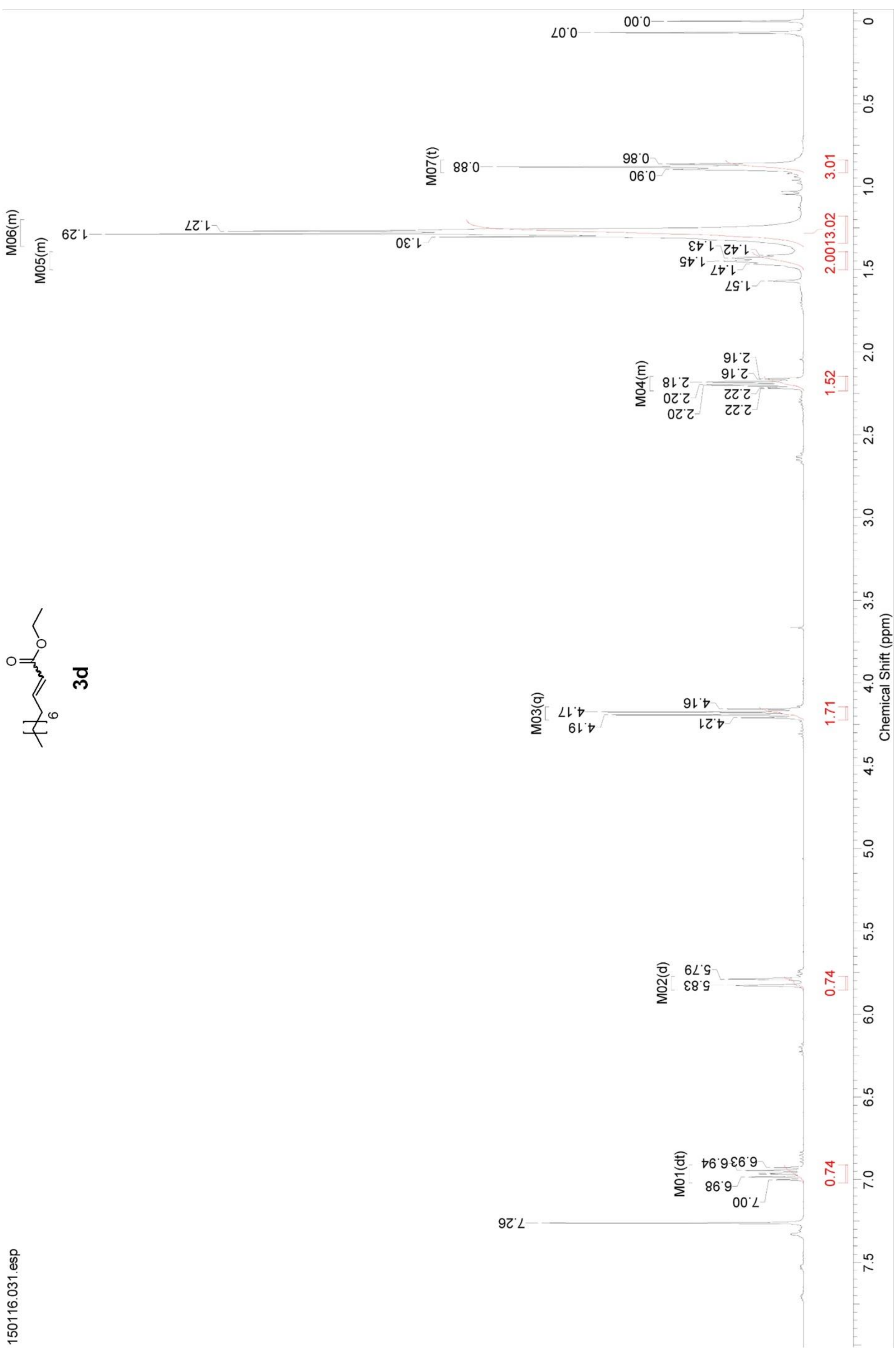
150116.031.esp
3d
6
O
M01(dt)
M02(d)
M03(q)
M04(m)
M05(m)
M06(m)
M07(t)
7.26
7.00
6.98
6.93
6.94
5.79
5.83
4.16
4.17
4.19
4.21
2.16
2.16
2.18
2.20
2.20
2.22
2.22
1.57
1.47
1.45
1.43
1.42
1.30
1.29
1.27
0.90
0.88
0.86
0.07
0.00
0.74
0.74
1.71
1.52
2.00
13.02
3.01
Chemical Shift (ppm)

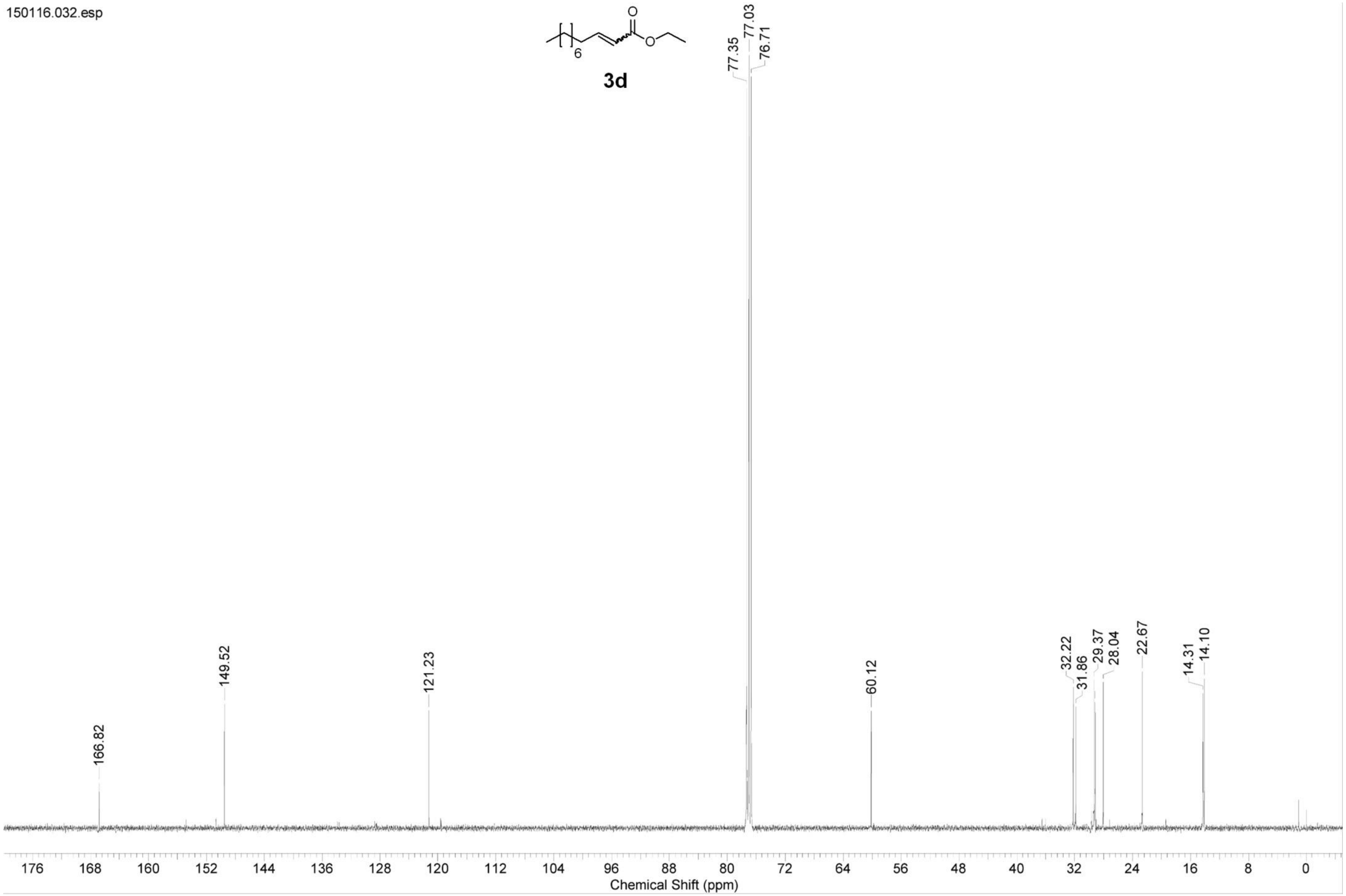

150116.032.esp
O
O
6
3d
77.35
77.03
76.71
166.82
149.52
121.23
60.12
32.22
31.86
29.37
28.04
22.67
14.31
14.10
176
168
160
152
144
136
128
120
112
104
96
88
80
72
64
56
48
40
32
24
16
8
0
Chemical Shift (ppm)

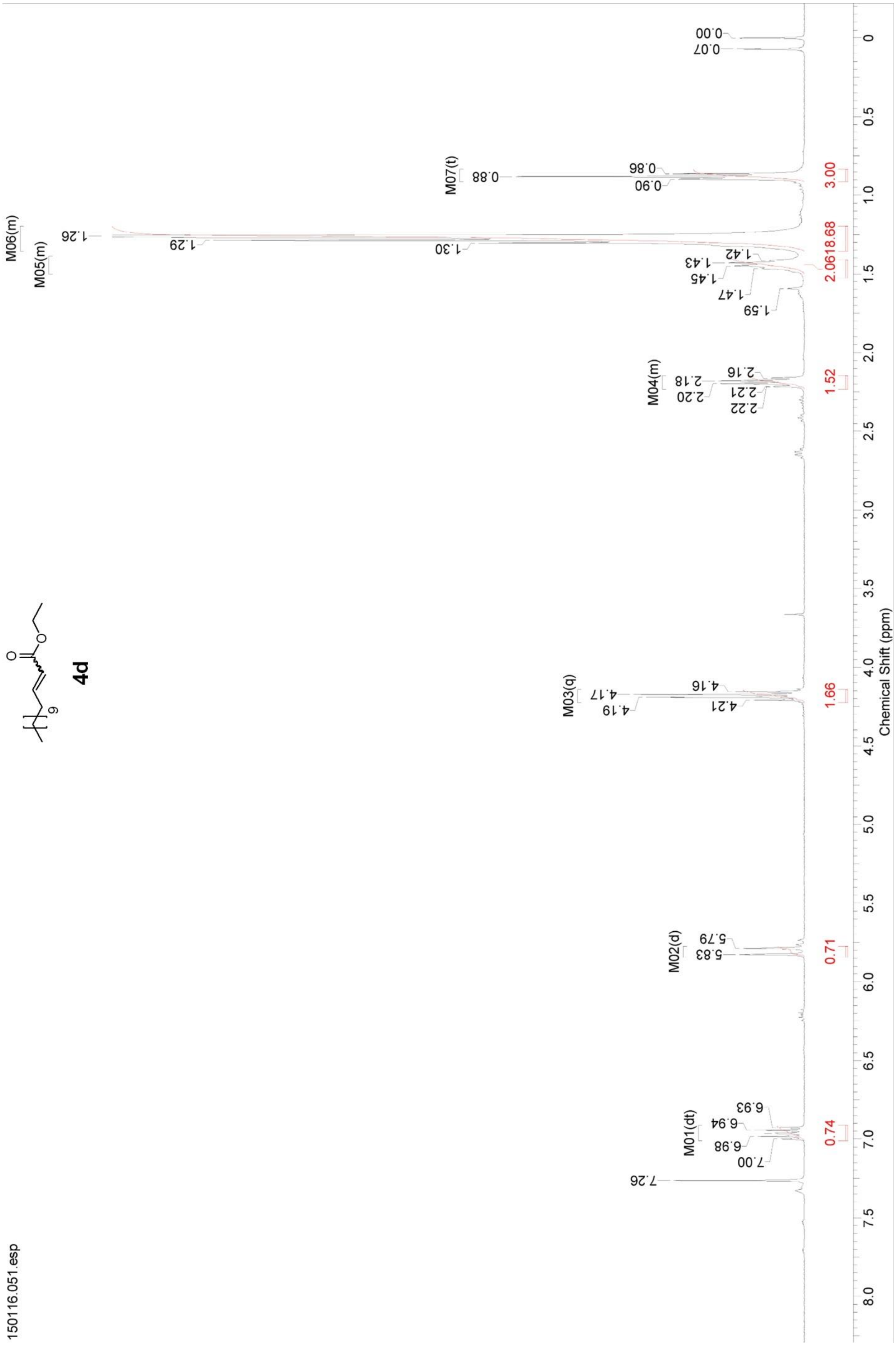
150116.051.esp
4d
9
O
M01(dt)
M02(d)
M03(q)
M04(m)
M05(m)
M06(m)
M07(t)
7.26
7.00
6.98
6.94
6.93
5.83
5.79
4.21
4.19
4.17
4.16
2.22
2.21
2.20
2.18
2.16
1.59
1.47
1.45
1.43
1.42
1.30
1.29
1.26
0.90
0.88
0.86
0.07
0.00
0.74
0.71
1.66
1.52
2.06
18.68
3.00
Chemical Shift (ppm)
8.0
7.5
7.0
6.5
6.0
5.5
5.0
4.5
4.0
3.5
3.0
2.5
2.0
1.5
1.0
0.5
0

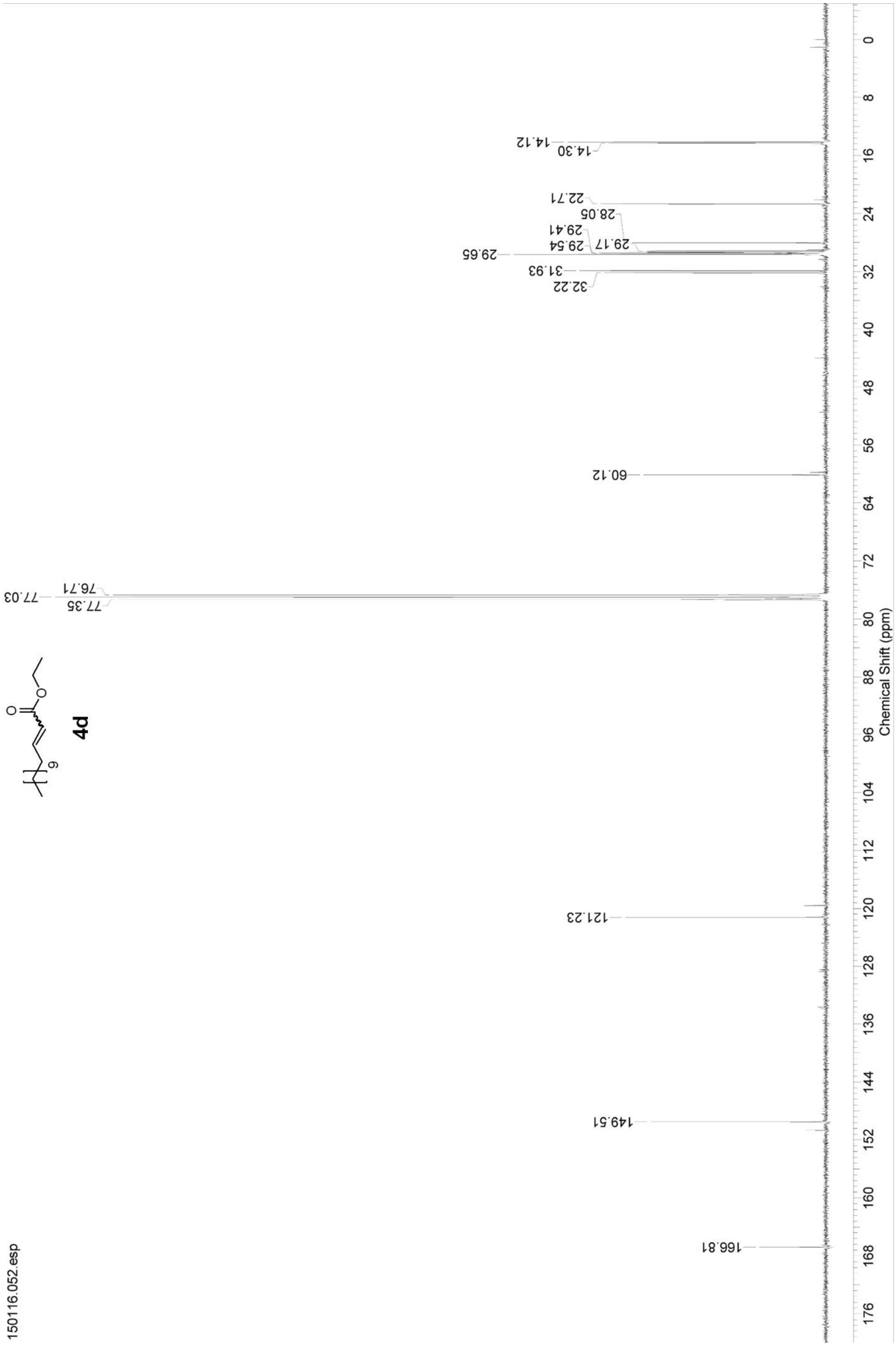

150116.052.esp
4d
166.81
149.51
121.23
77.35
77.03
76.71
60.12
32.22
31.93
29.65
29.54
29.41
29.17
28.05
22.71
14.30
14.12
Chemical Shift (ppm)

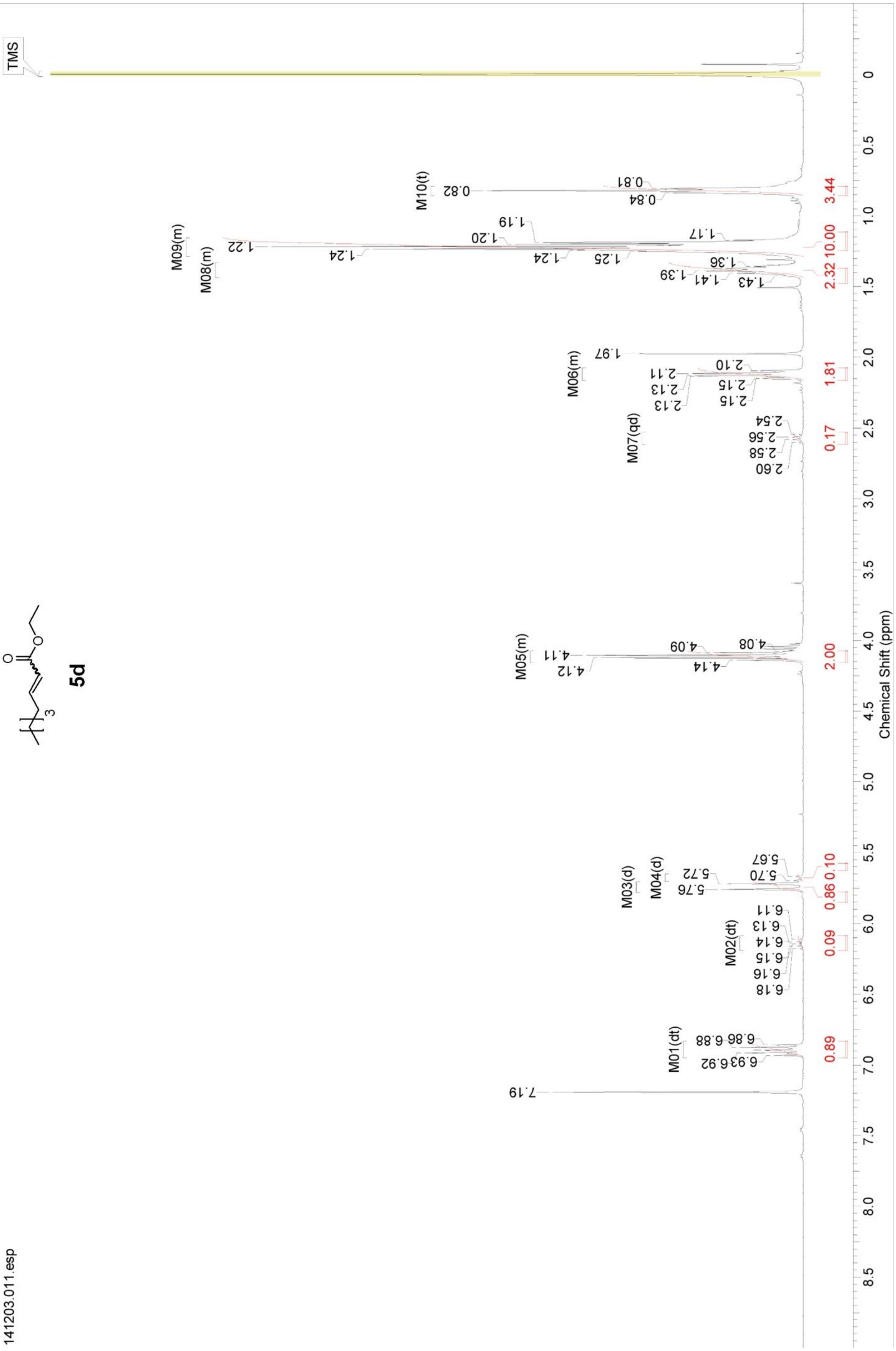

141203.011.esp
TMS
5d
Chemical Shift (ppm)

141203.012.esp

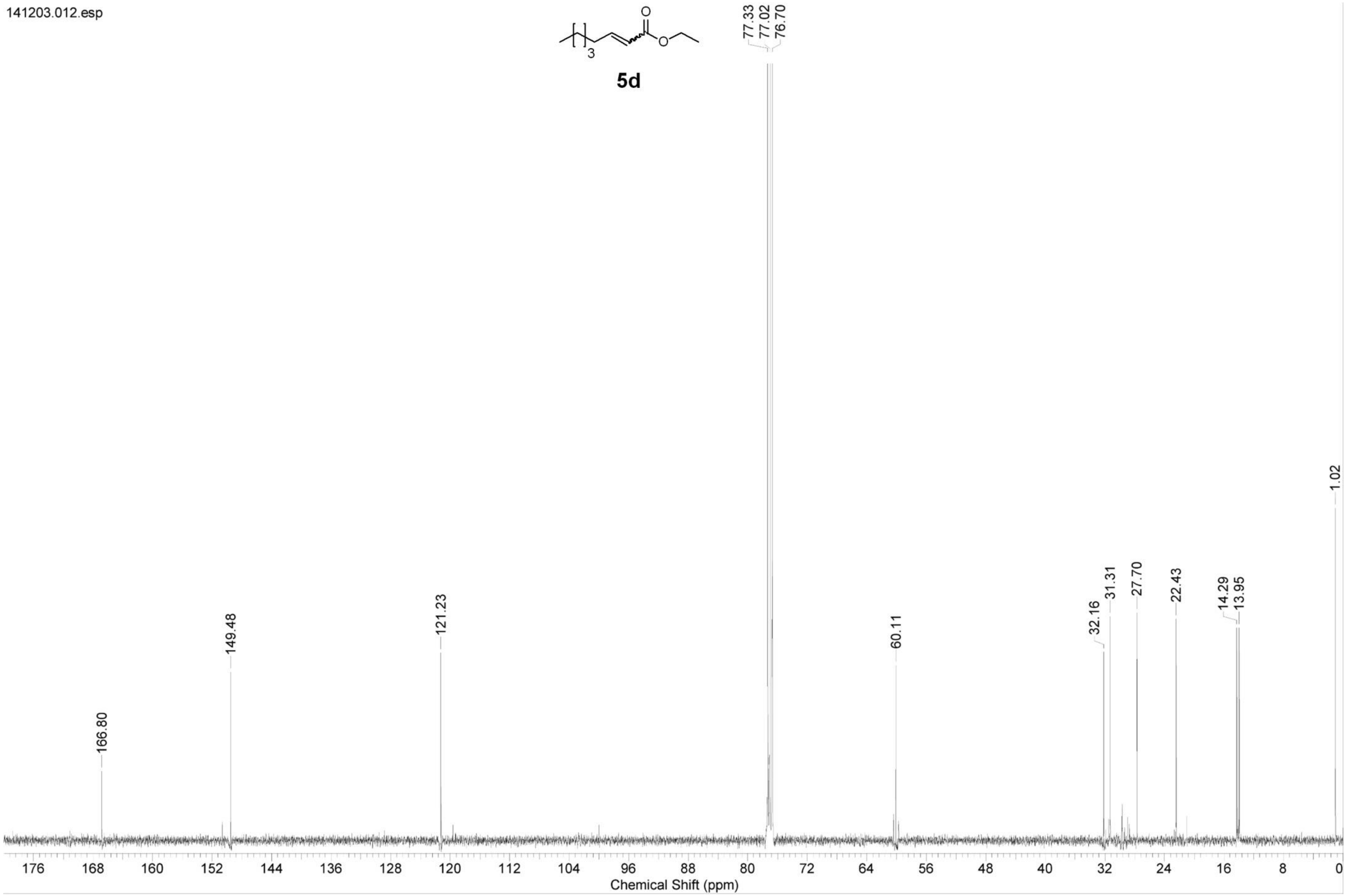


**5d**

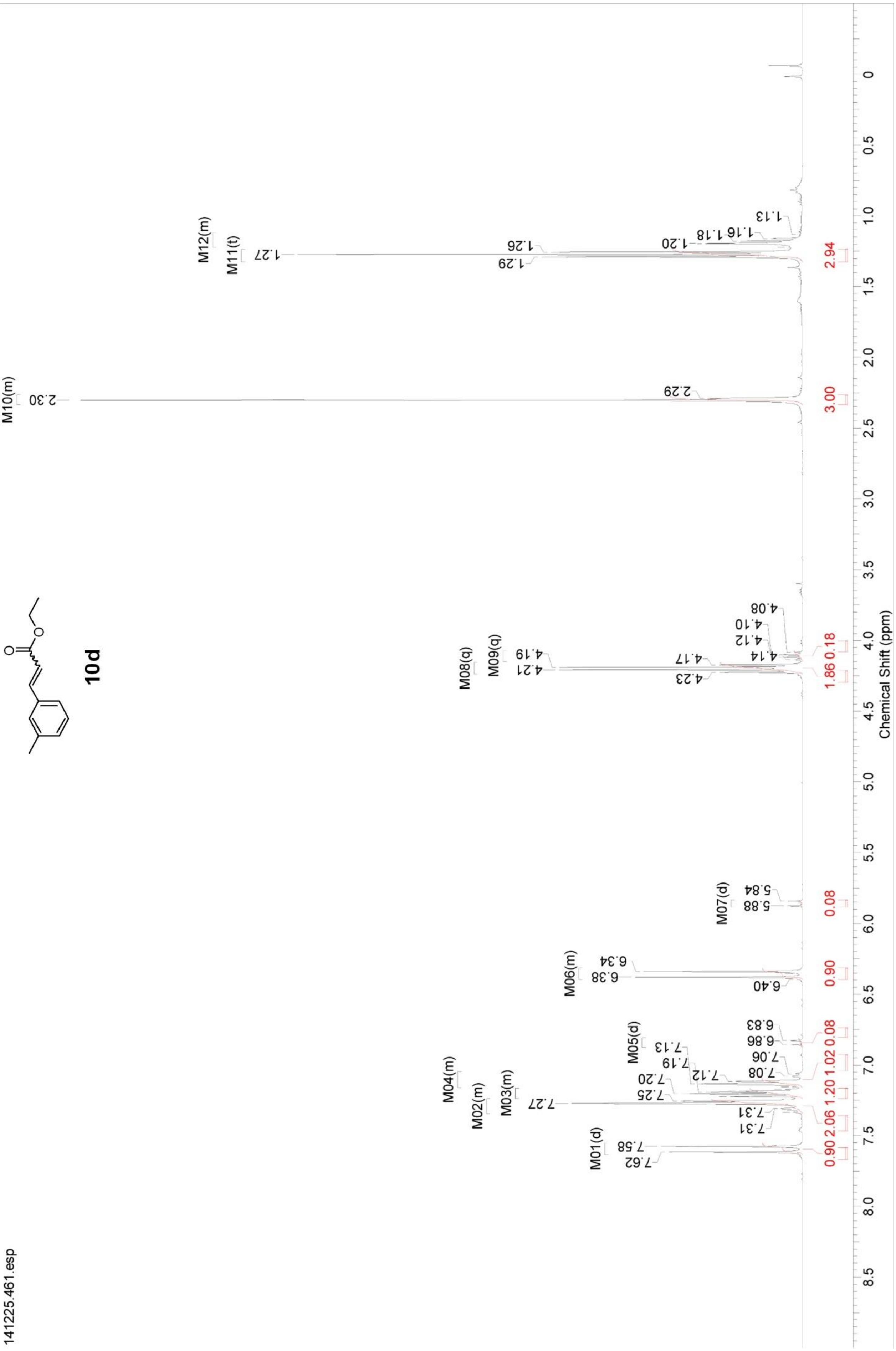
141225.461.esp
10d
M01(d) 7.58 7.62
M02(m)
M03(m) 7.27
M04(m)
7.25 7.20 7.19 7.13 7.12 7.31 7.31 7.08 7.06
M05(d) 6.86 6.83
M06(m) 6.34 6.38 6.40
M07(d) 5.84 5.88
M08(q) M09(q) 4.19 4.21 4.17 4.23 4.14 4.12 4.10 4.08
M10(m) 2.30 2.29
M11(t) 1.27 1.26 1.29
M12(m) 1.20 1.18 1.16 1.13
0.90 2.06 1.20 1.02 0.08 0.90 0.08 1.86 0.18 3.00 2.94
Chemical Shift (ppm)
8.5 8.0 7.5 7.0 6.5 6.0 5.5 5.0 4.5 4.0 3.5 3.0 2.5 2.0 1.5 1.0 0.5 0

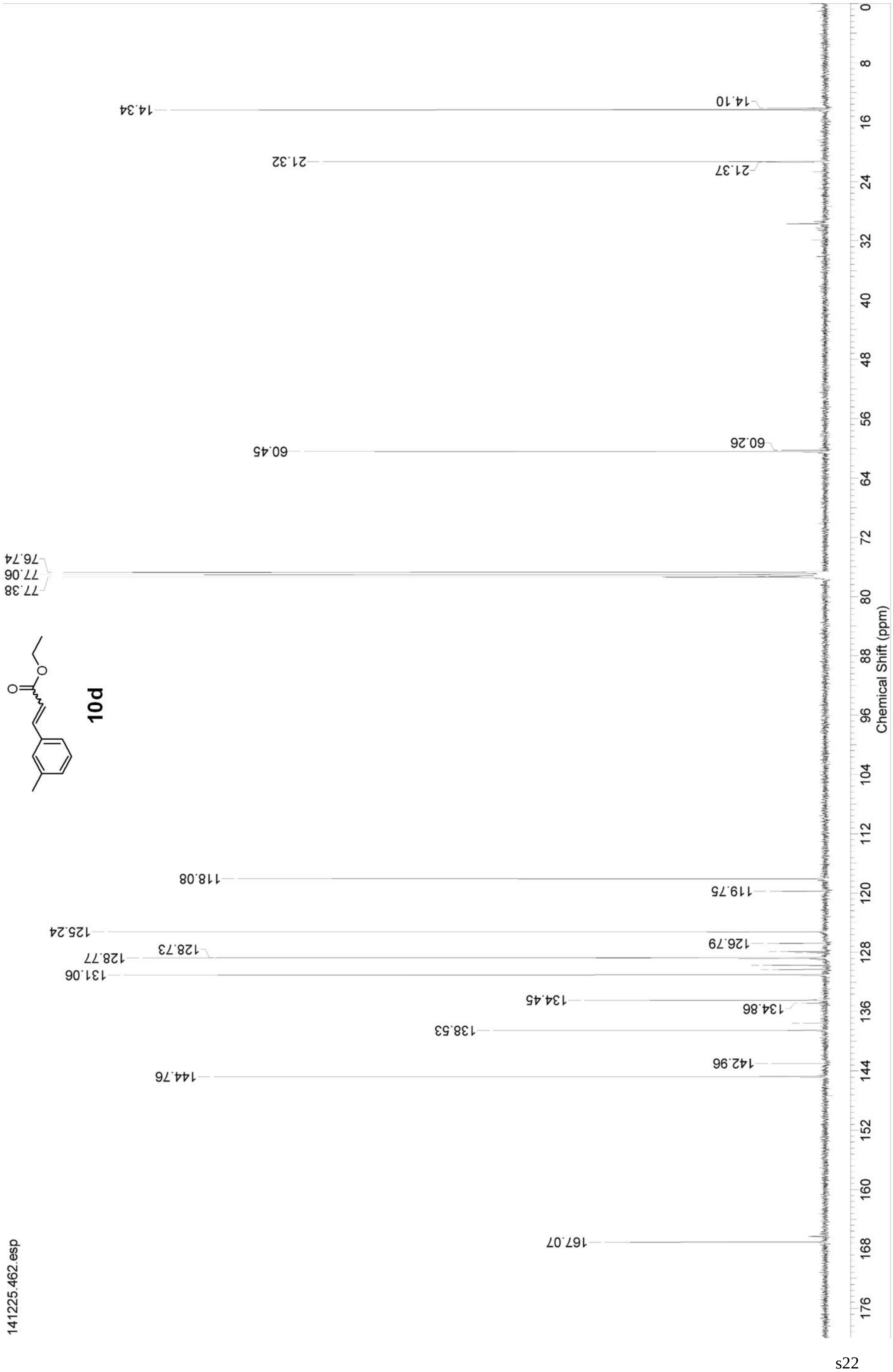
141225.462.esp
10d
Chemical Shift (ppm)
14.34
14.10
21.32
21.37
60.45
60.26
76.74
77.06
77.38
118.08
119.75
125.24
126.79
128.73
128.77
131.06
134.45
134.86
138.53
142.96
144.76
167.07

150128.441.esp

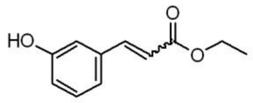


**12d**

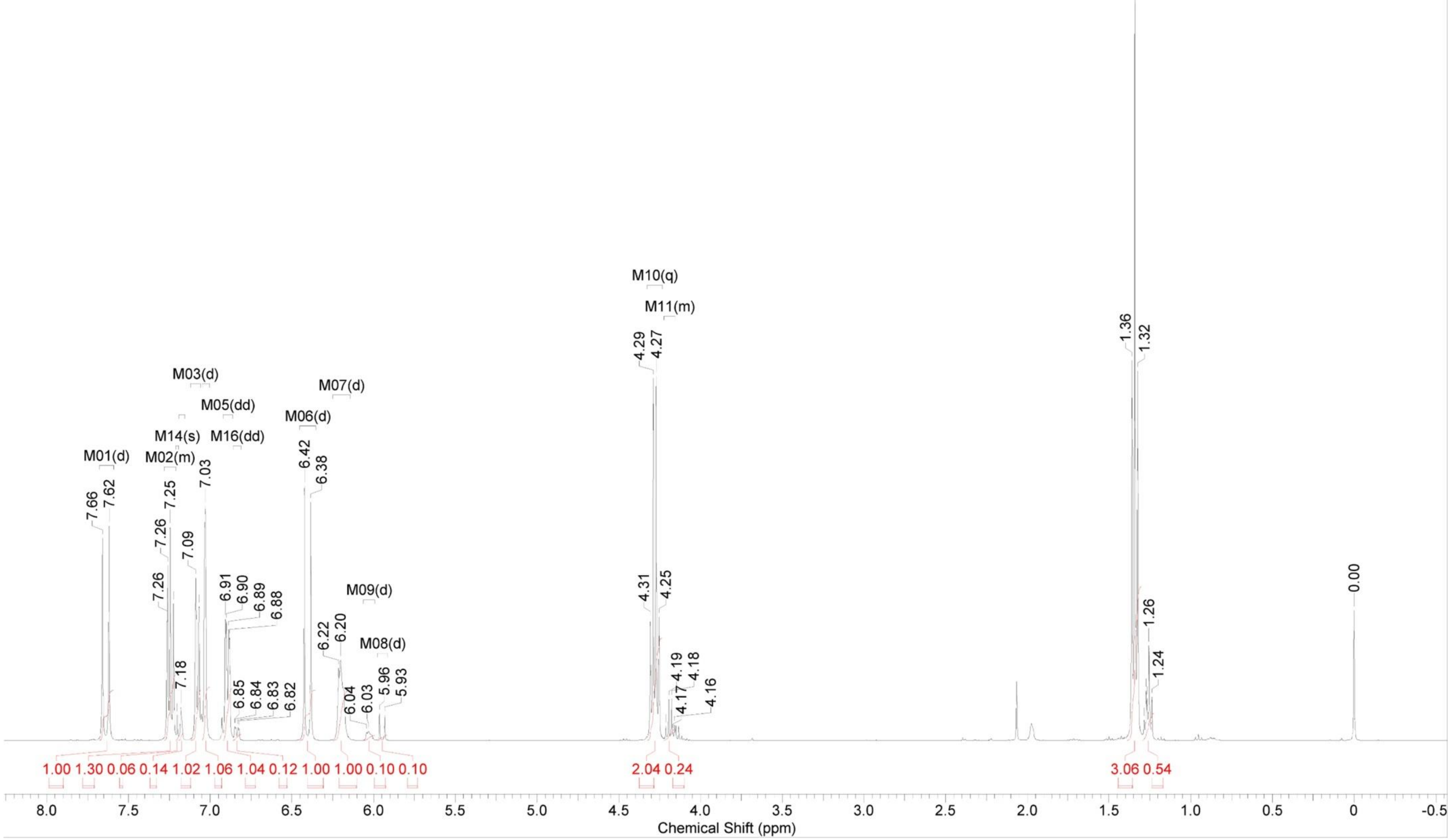

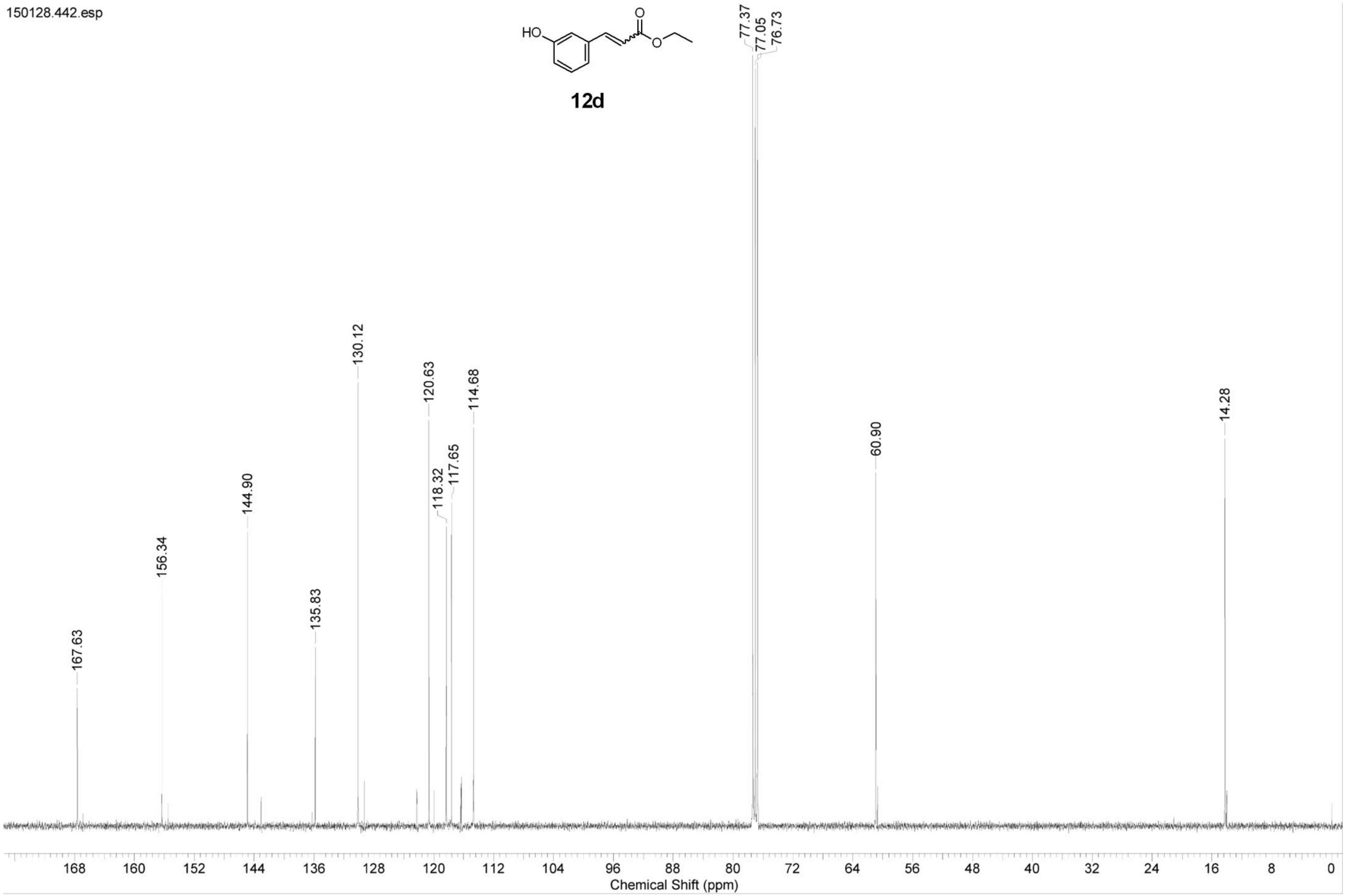
150128.442.esp
12d
77.37
77.05
76.73
167.63
156.34
144.90
135.83
130.12
120.63
118.32
117.65
114.68
60.90
14.28
168 160 152 144 136 128 120 112 104 96 88 80 72 64 56 48 40 32 24 16 8 0
Chemical Shift (ppm)

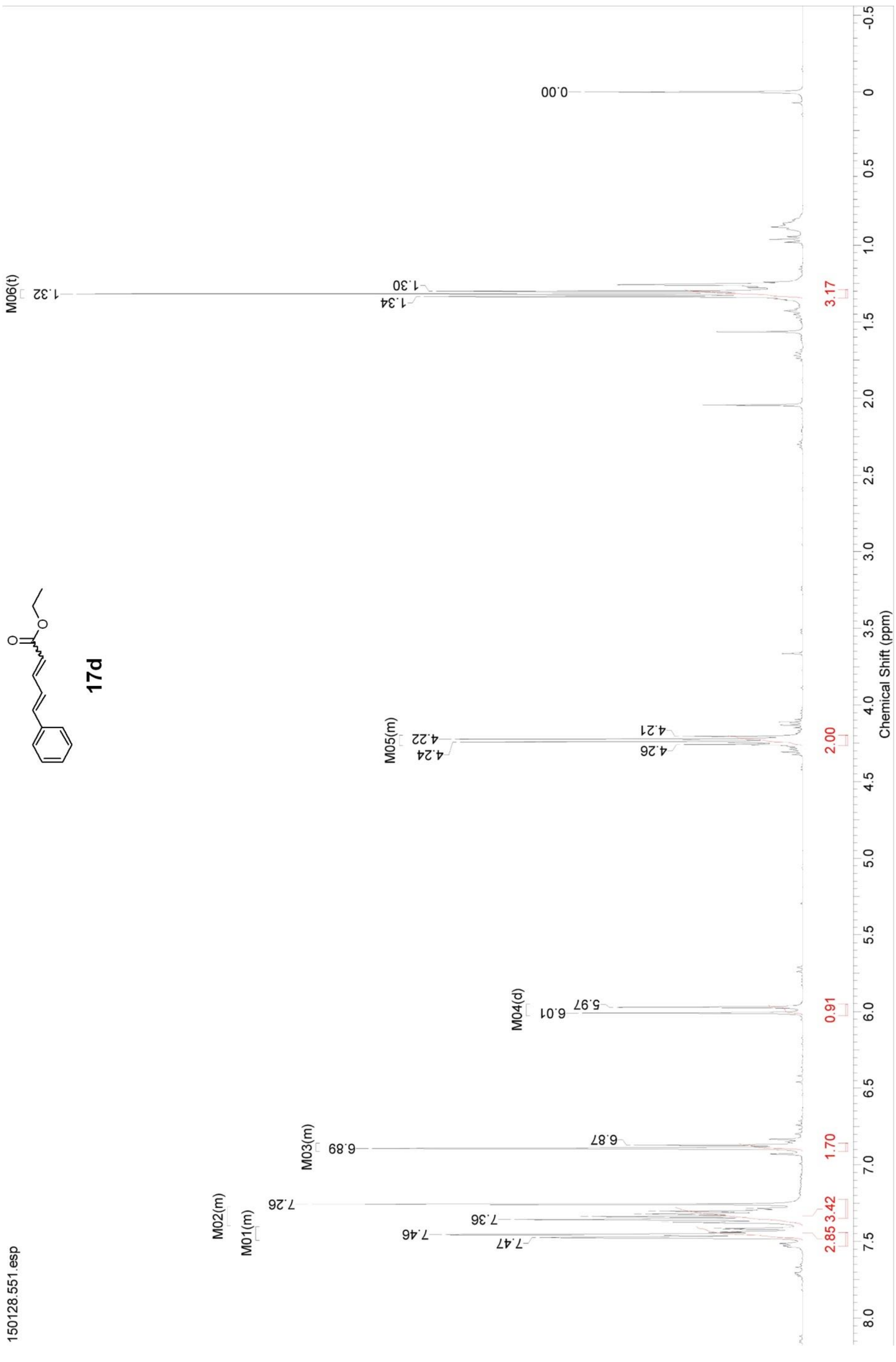
150128.551.esp
17d
M06(t)
1.32
1.30
1.34
0.00
M05(m)
4.22
4.24
4.21
4.26
M04(d)
5.97
6.01
M03(m)
6.89
6.87
M02(m)
M01(m)
7.26
7.36
7.46
7.47
3.17
2.00
0.91
1.70
3.42
2.85
Chemical Shift (ppm)

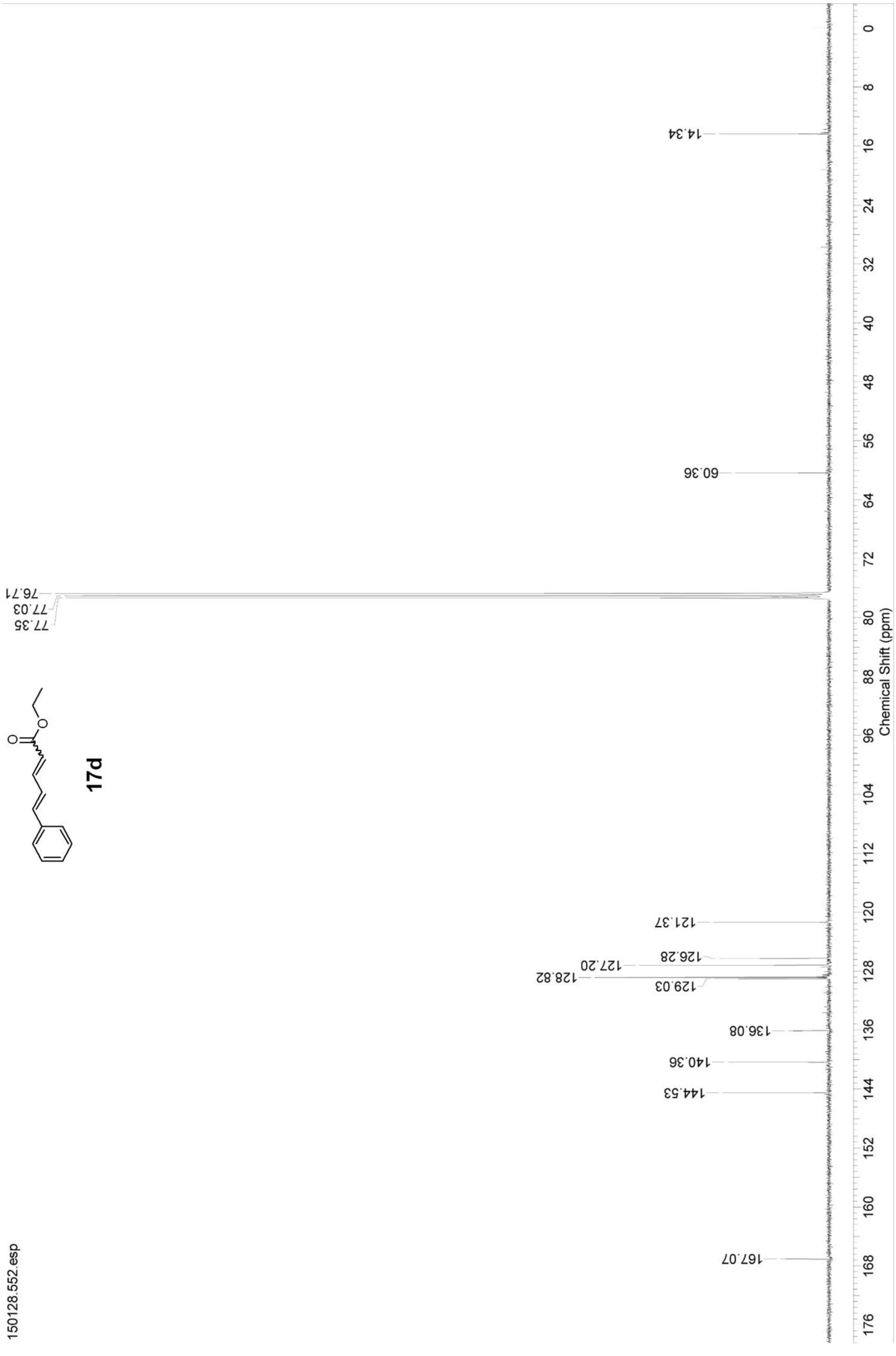
150128.552.esp
17d
14.34
60.36
76.71
77.03
77.35
121.37
126.28
127.20
128.82
129.03
136.08
140.36
144.53
167.07
Chemical Shift (ppm)
0
8
16
24
32
40
48
56
64
72
80
88
96
104
112
120
128
136
144
152
160
168
176

## References


1. He, A. M.; Li, T.; Daniels, L.; Fotheringham, I.; Rosazza, J. P. N. *Appl. Environ. Microbiol.* **2004**, *70*, 1874-1881. doi: 10.1128/Aem.70.3.1874-1881.2004
2. Shtratnikova, V. Y.; Bragin, E. Y.; Dovbnya, D. V.; Pekov, Y. A.; Schelkunov, M. I.; Strizhov, N.; Ivashina, T. V.; Ashapkin, V. V.; Donova, M. V. *Genome Announc*. **2014**, 2, 1e01177-13. doi: 10.1128/genomeA
3. Duan, Y.; Yao, P.; Chen, X.; Liu, X.; Zhang, R.; Feng, J.; Wu, Q.; Zhu, D. *J. Mol. Catal. B: Enzym.* **2015**, *115*, 1-7. doi: 10.1016/j.molcatb.2015.01.014
